# Cysteine dioxygenase 1 attenuates the proliferation via inducing oxidative stress and integrated stress response in gastric cancer cells

**DOI:** 10.1038/s41420-022-01277-x

**Published:** 2022-12-16

**Authors:** Gang Ma, Zhenzhen Zhao, Yang Qu, Fenglin Cai, Siya Liu, Han Liang, Rupeng Zhang, Jingyu Deng

**Affiliations:** 1 Department of Gastric Surgery, Tianjin Medical University Cancer Institute and Hospital, National Clinical Research Center for Cancer, Key Laboratory of Cancer Prevention and Therapy, Tianjin, P. R. China; 2 Tianjin’s Clinical Research Center for Cancer, Tianjin, 300060 P. R. China; 3 Department of Gastrointestinal Cancer Biology, Tianjin Medical University Cancer Institute and Hospital, National Clinical Research Center for Cancer, Key Laboratory of Cancer Prevention and Therapy, Tianjin, P. R. China

**Keywords:** Tumour-suppressor proteins, Stress signalling, Gastric cancer

## Abstract

Whereas cysteine dioxygenase 1 (CDO1) expression is lost due to its hypermethylated promoter across a range of cancer types including gastric cancer (GC), its functions and molecular underpinnings remain largely unknown. Here we demonstrate that reduced CDO1 expression is indicative of unfavorable prognosis in patients with GC. CDO1 overexpression in GC cells markedly inhibits cellular proliferation in vitro and in vivo. Mechanistically, CDO1 exerts this cytostatic effect via increasing oxidative stress and thus activating integrated stress response (ISR) in GC cells. High throughput screening (HTS) of antioxidants library identifies that Engeletin, a flavanonol glycoside, blunts oxidative stress and the ISR to relieve the inhibitory effect of CDO1 on the proliferation in GC cells. Additionally, genetic disruption or pharmaceutical inhibition of the ISR boosts the growth in the GC cells with CDO1 expression. Our data uncover the molecular mechanisms underlying the cytostatic function of CDO1 in the proliferation of GC cells.

## Introduction

It is becoming increasingly evident that deregulated expressions of metabolic enzymes contribute greatly to cancer initiation, progression, and metastasis. The deficiency of the cysteine dioxygenase 1 (CDO1) expression has been reported in lung [1], endometrial [2], breast [3], prostate [4], clear-cell renal cell [5], and gastrointestinal [6] cancers to date, and this aberration correlates with poor survival outcomes in patients with the examined cancer types [4, 5, 7–10]. Additionally, CDO1 shows promising diagnostic value in a few tumor types; for example, it could be an efficient strategy to determine minimal residual disease of the peritoneum in patients with gastric cancer (GC) [11, 12]. GC ranks the fifth for incidence and the fourth for cancer-caused mortality worldwide in 2020 [13]. CDO1 deficiency was found in GC tissues [14], suggesting that this protein probably contributes to GC progression.

CDO1 is essential in controlling intracellular cysteine homeostasis, as this enzyme converts cysteine into cysteine sulfinic acid (CSA), which is then catalyzed to taurine or sulfate (SO_4_^2−^) via two mutually exclusive pathways [15]. Genetic ablation of *Cdo1* in mice exhibited growth retardation and postnatal mortality [15]. Taurine level decreased markedly while plasma sulfate level increased slightly in *Cdo1*^-/-^ mice as compared with mice carrying one or two *Cdo1* alleles [15]. Notably, the sulfur chemicals generated during CDO1-catalyzed process probably disrupt redox balance and trigger oxidative stress. Ferroptosis is a regulated cell death program triggered by accumulated lipid-based reactive oxygen species [16]. Recently, CDO1 was found to be closely linked to erastin-induced ferroptosis in triple negative breast cancer cells, whereas silencing CDO1 expression blocked ferroptosis in GC cells [17, 18]. Moreover, loss of CDO1 accelerated proliferation via restraining the generation of the toxic intermediate product sulfite (SO_3_^2-^) in non-small cell lung cancer (NSCLC) cells with constitutively activated NRF2 [19]. Despite these advances, the overall mechanisms whereby CDO1 abrogates malignant phenotypes remain largely undefined.

Herein, we confirm that CDO1 expression was reduced in GC tissues and was an independent prognostic marker. Using in vitro and in vivo models, we show that enforced expression of CDO1 remarkably inhibited the proliferation in GC cells. Mechanistically, increased CDO1 impaired mitochondrial functions via inducing oxidative stress, which, in turn, triggered the integrated stress response (ISR). Our results uncover the molecular underpinnings of CDO1 in the suppression of cancer cell proliferation.

## Results

### Decreased CDO1 expression correlates with poor prognosis in patients with GC

**Fig. 1:**
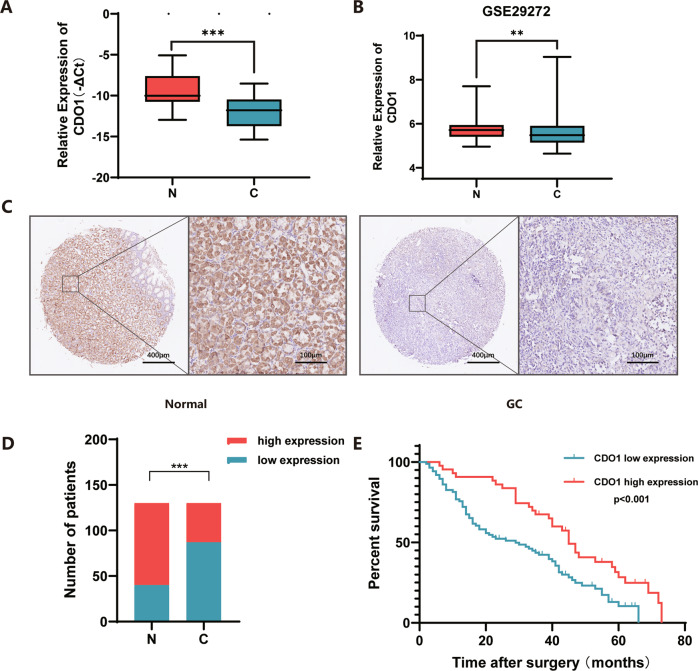
Reduced expression of *CDO1* in GC tissue is indicative of poor prognosis. **A** CDO1 mRNA level was examined in 30 paired GC and adjacent normal tissues, showing that it decreased in GC specimens relative to normal ones. **B** Analysis of CDO1 mRNA expression in one independent GC patient cohort (GSE29272) demonstrated that it was lower in GC samples than in normal counterparts. **C** IHC showed that CDO1 staining was stronger in normal gastric mucosa than in GC tissues. The representative images of one pair of tissues are presented here. Scale bars, 400 μm and 100 μm respectively. **D** Statistical analysis showed 50% of normal samples with high CDO1 protein expression while around 20% of GC tissues with high CDO1 protein level (*N* = 130). **E** Kaplan–Meier analysis of overall survival in GC patients with high or low CDO1 protein level showed that patients with higher CDO1 expression had better survival rate. The high or low expression of CDO1 in Kaplan–Meier analysis was stratified by median CDO1 protein level from IHC staining quantifications. **p < 0.01, ***p < 0.001.

To explore the role of CDO1 in GC, we firstly examined CDO1 expression in human GC and tumor-adjacent normal tissues. We found that CDO1 mRNA level markedly decreased in GC samples compared with adjacent normal tissues (*N* = 30, *p* < 0.001), which was consistent with the analysis result of CDO1 expression in a previous microarray dataset (GSE29272) from the Gene Expression Omnibus repository database (Fig. 1A, B). IHC assay demonstrated that GC samples exhibited lower CDO1 protein expression than the normal counterparts, as around 65% of the normal samples showed strong CDO1 staining while ~30% of GC samples were stained strongly (*N* = 130, *p* < 0.001) (Fig. 1C, D, Table 1). Correlation analysis uncovered that tumor sizes in patients with low CDO1 protein level were significantly larger than those with high CDO1 expression, suggesting that CDO1 deficiency probably supported hyperproliferation in GC cells (*p* = 0.0027; Table 2). Additionally, the overall survival (OS) rate in patients with GC was analyzed, showing that these patients with lower CDO1 level were susceptible to poorer survival (*N* = 130; median OS: high, 45 months vs. low, 29 months; *p* < 0.001) (Fig. 1E). More importantly, multivariate regression analysis validated that CDO1 was an independent prognostic factor for favorable OS in patients (*p* = 0.028; Table 3). Together, these results suggest that CDO1 played a critical role in GC progression.

**Table 1:** Statistic analysis of CDO1 expression in GC/normal tissues.

	CDO1		*χ*^2^-value	*P*-value
	Low expression	High expression		
**Normal**	40	90	34.00	<0.001
**Cancer**	87	43

**Table 2:** The clinical relevance of CDO1 expression in patients with GC.

Characteristics	CDO1		*χ*^2^-Value	*P*-value
	Low expression	High expression		
Gender				
Male	67	28	2.0701	0.1503
Female	20	15		
Age				
<60	41	26	2.050	0.1522
≥60	46	17		
Tumor size				
<6	25	24	8.9841	**0.0027**
≥6	62	19		
pT stage				
pT2	7	1	3.5902	0.1661
pT3	4	5		
pT4	76	37		
pN stage				
pN0	18	11	0.3973	0.5285
pN1-3	69	32		
Tumor location				
Upper third	6	10	4.4800	0.3450
Midle third	9	6		
Lower third	10	7		
More than2/3third All	5	2		
Lauren type				
Intestinal	67	29	3.7072	0.1567
Diffuse	29	11		
Mixed	1	3		

pT, pN and pTNM stage are defined using the American Joint Committee on Cancer (AJCC) Staging System, 8th Edition.

The significant *P*-values are in bold.

**Table 3:** Univariate/multivariate analysis of CDO1 expression in the outcome of patients with GC.

Characteristics	Univariate analysis		Multivariate analysis	
	HR (95%CI)	*P*-value	HR (95%CI)	*P*-value
Gender				
Male VS Female	1.512(0.991-2.308)	0.062		
Age				
≥60 VS < 60	1.474(0.996-2.183)	0.052		
Tumor size				
≥6 VS < 6	2.954(1.872-4.660)	**0.001**	2.507(1.570-4.004)	**0.01**
pT stage				
pT2 VS pT3-4	1.045(0.456-2.394)	0.917		
pN stage				
pN0 VS pN1-3	1.954(1.156-3.301)	**0.007**	01.533(0.896-2.621)	0.119
Expression of CDO1				
Low VS High	0.497(0.319-0.772)	**0.002**	0.603(0.384-0.946)	**0.028**

*HR* Hazard ratio; *CI* Confidence interval.

The significant *P*-values are in bold.

### CDO1 inhibits the proliferation in GC cells

**Fig. 2:**
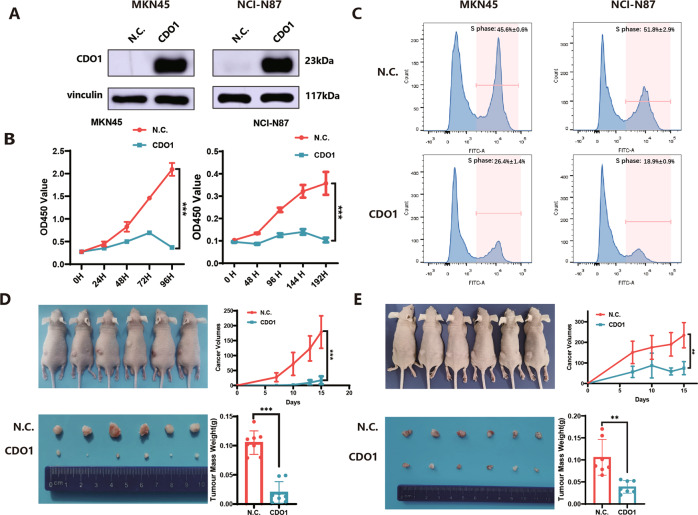
The inhibitory effect of CDO1 on the proliferation in GC cells. **A** The re-expression efficiency of CDO1 in MKN45 (left panel) and NCI-N87 (right panel) cells was verified by immunoblots. **B** Cellular growth of MKN45 (left panel) or NCI-N87 (right panel) cells from N.C. or CDO1-restored group was assayed using CCK-8 at the indicated time points respectively (*N* = 6, mean ± SD), showing that CDO1 remarkably suppressed the proliferation in these two GC cell lines. **C** MKN45 (left panel) or NCI-N87 (right panel) cells from N.C. or CDO1-restored group were stained with EdU and analyzed flow cytometry, showing that CDO1 markedly blocked the proliferation in GC cells. One representative result (*N* = 3, mean ± SD) are graphically presented here. **D**, **E** Tumor xenografts model based on female Balb/c nude mice demonstrated that the proliferation of MKN45 (**D**) and NCI-N87 (**E**) cells was substantially inhibited by CDO1 in vivo, as evidenced by the delayed growth at the indicated time intervals and the decreased weights of the tumor masses from MKN45 or NCI-N87 cells. **p < 0.01, ***p < 0.001.

Given that CDO1 expression was reduced in GC, we next sought to address the effect of CDO1 on cell propagation by transducing CDO1-expressing or control lentivirus into MKN45 and NCI-N87 cells, respectively. After the restored expressions of CDO1 were confirmed via immunoblots (Fig. 2A), CCK-8 assay was employed to determine how CDO1 influenced cellular proliferation. As shown in CCK-8 assay, CDO1 dramatically mitigated the viability of MKN45 or NCI-N87 cells, whereas the control cells grew gradually in the indicated time interval (Fig. 2B). Besides, EdU incorporation assay was used to measure replicating cells, showing that the number of the control MKN45 or NCI-N87 cells in S phase was significantly larger compared with those overexpressing CDO1 (Fig. 2C). Additionally, tumor masses derived from CDO1-expressing MKN45 or NCI-N87 cells were much smaller than those from the control cells, and the weights of the collected xenograft tumors were also dramatically decreased as the result of CDO1 restoration (Fig. 2D, E). Collectively, these findings demonstrate that CDO1 impaired the proliferation of GC cells in vitro and tumor growth in vivo.

### The anti-proliferation effect of CDO1 depends on its enzyme activity

**Fig. 3:**
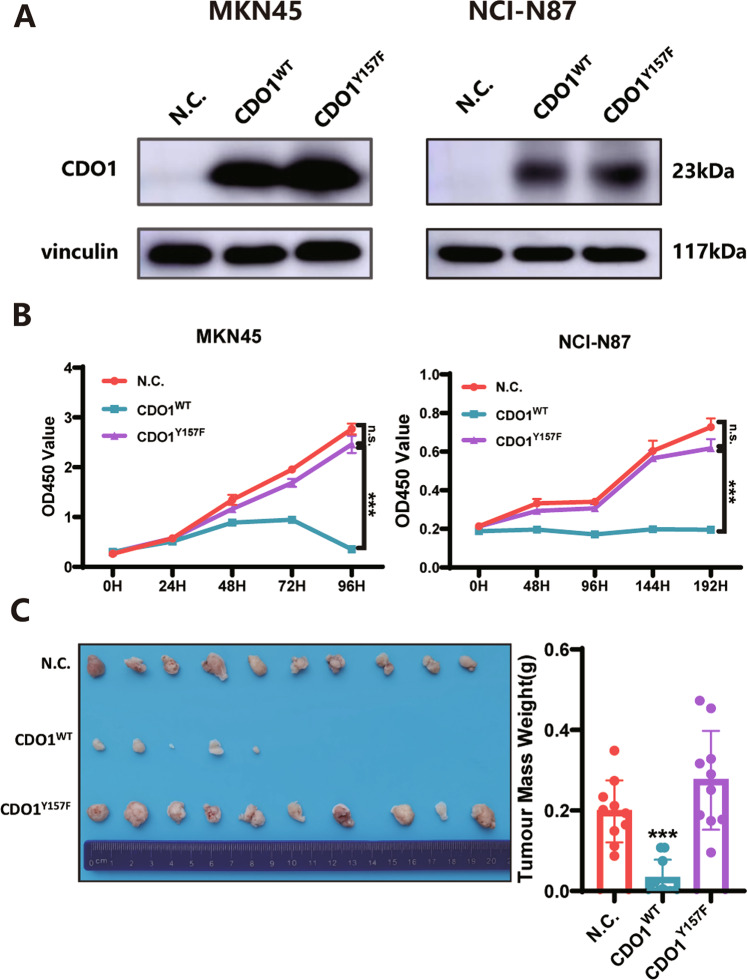
The anti-proliferation function of CDO1 is in an enzymatic activity-dependent manner. **A** The expression efficiency of CDO1^WT^ and CDO1^Y157F^ was confirmed via immunoblots in MKN45 (left panel) and NCI-N87 (right panel) cells. **B** CDO1^Y157F^, unlike CDO1^WT^, did not attenuate the growth of MKN45 (left panel) or NCI-N87 (right panel) cells in vitro, as indicated by CCK-8 assay at the indicated time points (*N* = 6, mean ± SD). **C** Tumor xenografts model showed that CDO1^Y157F^ could not inhibit the growth of MKN45 cells in vivo. The tumor masses from the indicated three groups were photographed and weighed (*N* = 10 mice per group). ***p < 0.001, n.s. means no significance.

We then attempted to investigate whether the anti-proliferation role of CDO1 relied on its enzyme activity, as some enzymes have been documented to exert non-canonical functions in cancers [20]. The mutation CDO1^Y157F^ without the catalytic activity, completely lost the inhibitory effect of CDO1 on the viability of MKN45 and NCI-N87 cells, as shown in CCK-8 assay (Fig. 3A, B). The colony formation potential was largely rescued in NCI-N87 cells with mutated, relative to wildtype, CDO1 (Supplementary Fig. 1). In line with these in vitro findings, CDO1^Y157F^-expressing MKN45 cells grew in the same manner as control cells did in immunocompromised mice (Fig. 3C), further confirming that this mutated CDO1 could not suppress proliferation. These results demonstrate that canonical enzyme activity of CDO1 was indispensable for its anti-proliferation function in GC cells.

### CDO1 exacerbates oxidative stress to suppress the proliferation in GC cells

**Fig. 4:**
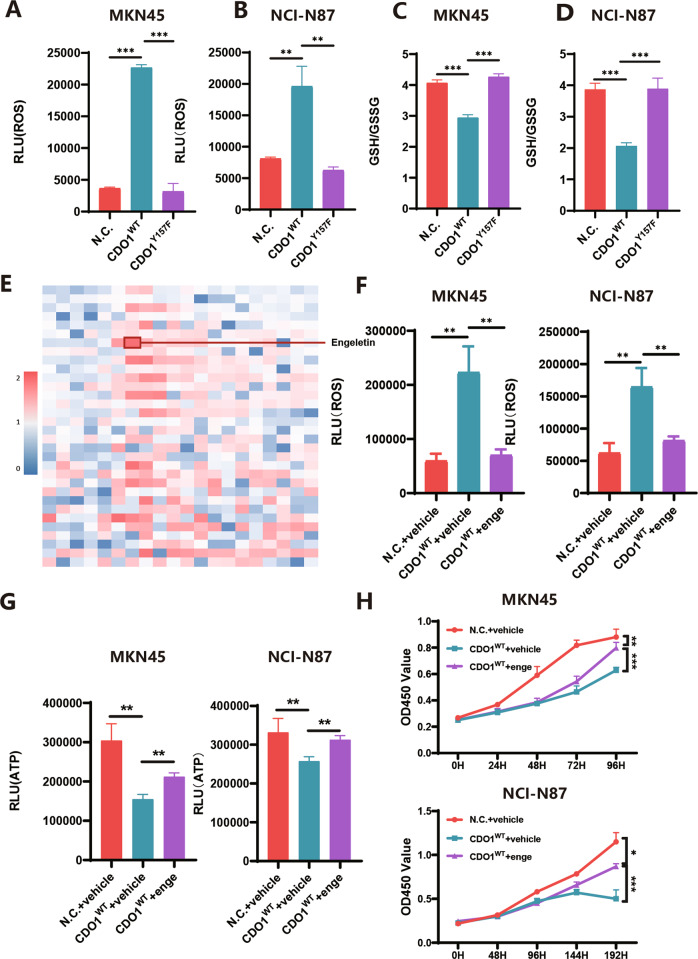
CDO1 induces oxidative stress in GC cells. **A**, **B** CDO1^WT^ markedly increased ROS level while CDO1^Y157F^ did not alter ROS production in MKN45 (**A**) and NCI-N87 (B) cells. **C**, **D** CDO1^WT^ rather than CDO1^Y157F^ decreased GSH/GSSG ratio in MKN45 (**C**) and NCI-N87 (**D**) cells. **E** Heatmap of the fold changes in all antioxidants subjected to HTS relative to MKN45 cells treated with DMSO (Vehicle). Intracellular ATP level, measured using CellTiter-Glo, was used as the surrogate of viable cells. Luminescent reads of engeletin-treated MKN45 cells approximately increased by two times. The fold changes were measured as: Fold change = (Reads _(antioxidant)_-Reads _(Vehicle)_) / Reads _(Vehicle)_. **F** Engeletin-treated MKN45 (left panel) or NCI-N87 (right panel) cells with CDO1^WT^ restoration displayed reduced ROS production compared to these cells exposed to vehicle. **G** Treatment of Engeletin in MKN45 (left panel) or NCI-N87 (right panel) cells with restored CDO1^WT^ increased ATP generation relative to vehicle-treated cells. **H** CCK-8 assays demonstrated that engeletin treatment relieved the inhibition of CDO1^WT^ on the proliferation in MKN45 (upper panel) or NCI-N87 (bottom panel) cells in vitro. *p < 0.05, **p < 0.01, ***p < 0.001.

A recent report showed that cysteine sulfinic acid (CSA) and sulfite (SO_3_^2-^), the byproducts of CDO1-mediated metabolism, were detrimental to the viability in non-small cell lung cancer cells [19]. Thus, we asked if CDO1 enhanced oxidative stress in GC cells. Reactive oxygen species (ROS) level markedly increased in MKN45 or NCI-N87 cells with CDO1 overexpression (Fig. 4A, B). In addition, CDO1 significantly reduced glutathione (GSH) level while increased the amount of oxidized glutathione (GSSG), thereby decreasing the ratio of GSH against GSSG in GC cells (Fig. 4C, D). On the other hand, CDO1^Y157F^ failed to trigger accumulation of ROS or decreased GSH/GSSG ratio compared with CDO1^wt^ in GC cells (Fig. 4A–D).

In order to determine if elevated oxidative stress was primarily responsible for this cytostatic effect, N-acetylcysteine (NAC) was used to relieve the oxidative stress induced by CDO1. However, we did not observe that the proliferation and the ATP production was restored in GC cells treated with NAC relative to vehicle-treated counterparts (Supplementary Fig. 2A, B). Given that oxidative stress is multifactorial, we then used HTS of antioxidant compound library to identify the antioxidants that could mitigate the CDO1-induced oxidative stress, showing that several compounds, such as Engeletin, could relieve CDO1-induced inhibition on the proliferation in MKN45 cells (Fig. 4E). Engeletin is a flavanonol glycoside extracted from *hymenaea martiana*, and its antioxidant function has been uncovered recently [21, 22]. Engeletin administration reduced the level of ROS, rescued ATP generation, and increased the viability in GC cells with CDO1 overexpression (Fig. 4F–H). These findings indicate that CDO1 attenuated the propagation by eliciting oxidative stress in GC cells.

### CDO1-induced oxidative stress impairs mitochondrial respiration function

**Fig. 5:**
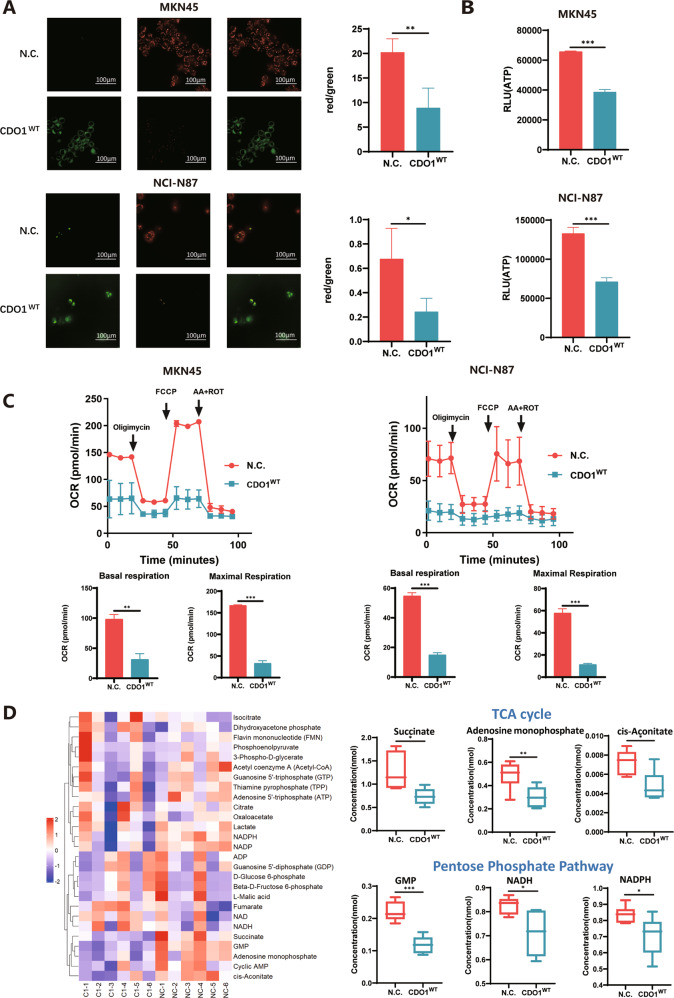
CDO1 restoration impairs mitochondrial function. **A** JC-1 staining was performed as described in Materials and Methods. The representative images of JC-1 staining in the control or the CDO1-restored MKN45 or NCI-N87 cells are presented here (left panel). The fluorescence intensity ratio of JC-1 aggregates (red) against JC-1 monomers (green) markedly decreased in MKN45 and NCI-N87 cells, indicating that mitochondrial depolarization occurred in GC cells with CDO1^WT^ re-expression. Scale bars, 100 μm. **B** ATP generation, which is the major function of mitochondria, was substantially reduced as the result of CDO1^WT^ restoration. **C** Test of OCR in MKN45 (left panel) and NCI-N87 (right panel) cells from the N.C. and CDO1^WT^ group, showing that respiration activity of mitochondria was markedly blocked by CDO1^WT^. Bottom, quantification of basal respiration and maximal respiration as measured by OCR (mean ± SD; *N* = 3). Data shown is a representative experiment of two independent ones. **D** Metabolomics study targeting key metabolites implicated in cellular ATP generation process showed that succinate, AMP, cis-aconitate, GMP, NADH, and NADPH were all significantly decreased in CDO1^WT^-overexpressing MKN45 cells compared to N.C. ones. *p < 0.05, **p < 0.01, ***p < 0.001.

Oxidative stress impairs almost all essential biological processes including, in particular, mitochondrial functions [23]. Mitochondrial membrane potential was measured using JC-1 dye, showing that control GC cells displayed the red fluorescence of JC-1 precipitates while the green fluorescence of JC-1 monomers were primarily observed in those with restored CDO1 expression (Fig. 5A). CDO1 also reduced ATP production, as the surrogate of mitochondrial integrity, by ~50% in MKN45 and NCI-N87 cells (Fig. 5B). Seahorse analysis showed that CDO1 markedly inhibited the aerobic respiration in GC cells by reducing OCR of both basal and maximal respiration (Fig. 5C). Additionally, we found that CDO1 remarkably reduced the amount of the glucose uptake and of the secreted lactate in both MKN45 and NCI-N87 cells (Supplementary Fig. 3A, B). As mitochondria function as the hub of cellular energy metabolism, we thereby examined the key metabolites in this process (Supplementary Table 3), finding that succinate, adenosine monophosphate (AMP), cis-aconitate, guanosine 5'-monophosphate (GMP), nicotinamide adenine dinucleotide (NADH), and nicotinamide adenine dinucleotide phosphate (NADPH) significantly decreased in CDO1-restored GC MKN45 cells (Fig. 5D). Notably, NADPH maintains the level of the reduced glutathione (GSH) and thioredoxins (TXN) [24], and the reduction in NADPH amount further confirmed that CDO1 triggered a more severe oxidative stress in cells. Together, these results show that the imbalanced redox state that was triggered by CDO1 hampered the function of mitochondria.

### CDO1-triggered oxidative stress activates the ISR in GC cells

**Fig. 6:**
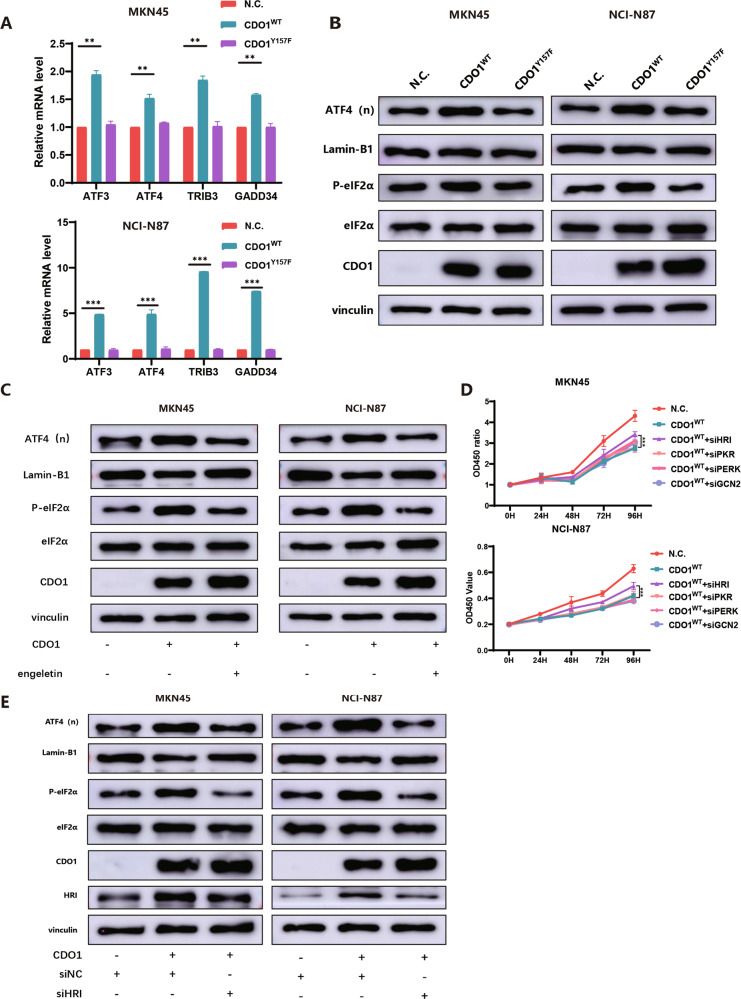
CDO1-induced oxidative stress triggers ISR. **A** The expression of several key downstream genes of ISR, such as *ATF3*, *ATF4*, *TRIB3* and *GADD34*, was examined by qPCR, demonstrating that CDO1^WT^, rather than CDO1^Y157F^, upregulated the mRNA expression of these genes in MKN45 (upper panel) and NCI-N87 (bottom panel) cells. **B** Immunoblots showed that ATF4 accumulated in the nucleus and phosphorylated eIF2α (Ser51) level in the cytoplasm increased in MKN45 (left panel) and NCI-N87 (right panel) cells with CDO1^WT^ re-expression. However, CDO1^Y157F^ did not activate ISR. **C** Engeletin reduced the nuclear accumulation of ATF4 and cytoplasmic increase in phosphorylated eIF2α (Ser51), which were induced by CDO1^WT^, in MKN45 (left panel) and NCI-N87 (right panel) cells. **D** HRI knockdown relieved the CDO1^WT^-induced suppression on the proliferation in MKN45 and NCI-N87 cells in vitro. **E** Immunoblots showed that HRI knockdown decreased nuclear expression of ATF4 and cytoplasmic level of phosphorylated eIF2α (Ser51) in CDO1^WT^-restored MKN45 (left panel) and NCI-N87 (right panel) cells. Lamin-B1 was used as the loading control for nuclear proteins and Vinculin for cytoplasmic proteins, respectively. *p < 0.05, **p < 0.01, ***p < 0.001.

ISR is an evolutionally conserved signaling pathway in response to diverse intra- and extra-cellular stresses such as oxidative stress [25]. Thus, we examined whether CDO1 triggered ISR in GC cells. Firstly, we tested the mRNA expression of the downstream effectors of ISR, finding that the mRNA level of *ATF3*, *ATF4*, *TRIB3* and *GADD34* significantly increased in GC cells with CDO1 overexpression compared with control ones (Fig. 6A). However, inactive CDO1 failed to change the expression of ISR-related genes (Fig. 6A). Then, we observed that the nuclear accumulation of ATF4 and the level of phosphorylated eIF2α (Ser51), which are two molecular hallmarks of activated ISR, increased, while Engeletin administration reduced the level of nuclear ATF4 and cytoplasmic phosphorylated eIF2α (Ser51) in CDO1^WT^-restored GC cells (Fig. 6B, C). Four kinases, including heme-regulated eIF2α kinase (HRI), PKR-like ER kinase (PERK), double-stranded RNA-dependent protein kinase (PKR) and general control nonderepressible 2 (GCN2), are responsible for sensing stresses and relaying signals by phosphorylating eIF2α (Ser51) in ISR [25]. We found that HRI knockdown, rather than the deficiency of the other three kinases, promoted the proliferation in GC cells with CDO1 restoration (Fig. 6D, Supplementary Fig. 4A, B). Moreover, HRI deficiency mitigated ISR activation caused by CDO1 in GC cells, indicating that HRI is the kinase to mediate CDO1-induced ISR in this study (Fig. 6E).

### The cytostatic effect of CDO1 is dependent on the ISR in GC cells

**Fig. 7:**
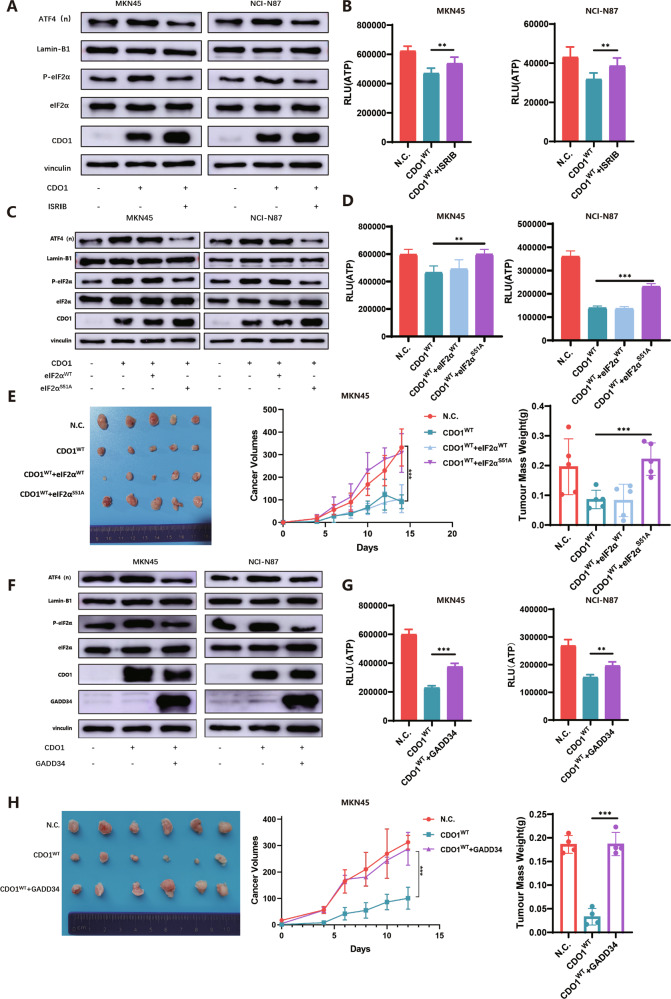
The inhibitory effect of CDO1 on the proliferation in GC cells relies on ISR. **A**, **B** ISRIB, a small inhibitor of ISR, was added in N.C. and CDO1^WT^ GC cells, showing that it attenuated ISR activation by reducing the nuclear level of ATF4 and cytoplasmic level of phosphorylated eIF2α (Ser51) (**A**). ATP production was increased in response to ISRIB treatment in CDO1^WT^-restored MKN45 (left panel) and NCI-N87 (right panel) cells (**B**). **C**, **D** The Ser51 was mutated to Ala51 to generate the activity-dead eIF2α (eIF2Α^S51A^). Immunoblots showed that eIF2Α^S51A^, compared to eIF2Α^WT^, remarkably blocked the activation of ISR (C) and, as uncovered by luminescent assay, increased ATP generation to some extend (**D**) induced by CDO1^WT^ in GC cells. Left panel, MKN45 cells; right panel, NCI-N87 cells. **E** Subcutaneous tumor xenograft model showed that eIF2Α^S51A^ markedly relieved the inhibition of CDO1^WT^ on the growth of MKN45 cells in Balb/c nude mice (*N* = 5 per the indicated group). **F**, **G** GADD34 was expressed in MKN45 (left panel) and NCI-N87 (right panel) cells with overexpressed CDO1, showing that GADD34 partially suppressed ISR activation (**F**) and restored ATP production (**G**). **H** GADD34 markedly restored the growth of MKN45 cells in vivo (*N* = 6 per the indicated group). Lamin-B1 was used as the loading control for nuclear proteins and Vinculin for cytoplasmic proteins, respectively. **p < 0.01, ***p < 0.001.

As CDO1 elicited ISR in GC cells, we asked if its anti-proliferation effect was attributable to this signaling pathway. Pharmaceutical inhibition of ISR using ISRIB restored ATP production in CDO1-overexpressed GC cells (Fig. 7A, B). Since phosphorylation of Ser51 in eIF2α is the key event of ISR activation, we delivered eIF2α^WT^ or eIF2Α^S51A^ into CDO1-restored MKN45 cells respectively to test the potential influence of mutated eIF2Α on cellular viability. eIF2Α^S51A^ markedly deactivated ISR and increased ATP generation in GC cells (Fig. 7C, D). Meanwhile, we found that the tumor masses derived from MKN45 cells with eIF2Α^S51A^ and CDO1^WT^ expression simultaneously were larger than counterparts in CDO1^WT^/eIF2Α^WT^ group (Fig. 7E). Likewise, as a naturally occurred inhibitor of ISR, GADD34 was introduced into MKN45 cells in the presence of CDO1^WT^, showing that ATP amount increased relative to cells expressing CDO1^WT^ only (Fig. 7F, G). Furthermore, the growth of MKN45 cells in vivo was also boosted due to GADD34 overexpression (Fig. 7H). Collectively, these data corroborate that ISR is the major mechanism underlying the cytostatic effect of CDO1 in GC cells.

## Discussion

The deficiency in CDO1 expression in multiple cancer types implicates its anti-cancer functions. For example, CDO1 expression was preferably silenced due to hypermethylated promoter in NSCLC cells with KEAP1 mutation, thereby protecting cells from oxidative damages through NRF2-mediated antioxidant defense; while its restoration remarkably antagonized the viability in NSCLC cells by producing toxic sulfur-based chemicals or depleting intracellular NADPH [19]. Likewise, our results show that CDO1, whose protein level was markedly decreased in GC samples and a panel of GC cell lines, also substantially attenuated the proliferation in GC cells. Additionally, mutated CDO1 without enzymatic activity did not exert the anti-proliferative effect on GC cells, indicating that CDO1-regulated metabolic process is the basis underlying its function as it does in NSCLC cells. Of note, GC cells is probably not dependent on NRF2/KEAP1-mediated antioxidant mechanisms, as GC incidence in *Nrf2*-null mice did not significantly increase relative to *Nrf2*-wildtype littermates, when they were all fed with benzo(a)pyrene [26]. Thus, we further explore the molecular mechanisms by which CDO1 blunted the proliferation in GC cells.

The roles of oxidative stress in cancer are very perplexing due to variable cancer settings, distinct intensity and duration of oxidative stress, as well as specific stages of cancer evolution [24]. A prospective study shows that oxidative stress in patients with GC and their first-degree relatives significantly increased compared to dyspeptic patients [27], indicating that elevated oxidative stress probably contributes greatly to gastric carcinogenesis. *Helicobacter pylori* infection evokes long-lasting oxidative stress, which, in turn, fosters malignant transformation through various mechanisms [28]. For example, oxidative stress induced by *Helicobacter pylori* infection increased histone H3 acetylation in the promoter of *capping actin protein of muscle Z-line α subunit 1* (*CAPZA1*) to boost its expression [29]. CAPZA1 promoted the expression of *CD44* and *epithelial splicing regulatory protein 1* (*ESRP1*), the latter which spliced CD44 into CD44 variant 9 (CD44v9), a marker of gastric cancer stem-like cells [29]. In order to sustain the enhanced intracellular oxidative level in favor of hyperproliferative phenotype, GC cells exploit enzymes to generate more NADPH. Recent studies uncovered that depletion of nicotinamide nucleotide transhydrogenase (NNT), malic enzyme 1 (ME1), or diacylglycerol acyltransferase 2 (DGAT2) reduced NADPH level, further promoting apoptosis and undermining metastasis [30–32]. Together, these results underscore the proper control of redox homeostasis in influencing initiation, progression and metastasis in GC cells. In line with the previous findings in NSCLC cells, CDO1 restoration in GC cells elicited oxidative stress as evidenced by elevated ROS, reduced GSH/GSSG and NADPH, as well as damages to respiratory function of mitochondria *et al*. However, we did not observe that CDO1 triggered cellular death, such ferroptosis, in GC cells (Supplementary Fig. 5). Intriguingly, NAC treatment failed to subdue the cytostatic effect of oxidative stress on GC cells and to increase ATP production, given that it is well documented that NAC scavenges ROS under various conditions. This may be because the SO_3_^2-^ produced by CDO1, in addition to depleting cysteine (Cys) via forming Cys-SO_3_^2-^, plays pleiotropic roles in GC cells. High-throughput antioxidants screening showed that several drugs, including Engeletin, which usually deactivate NF-κB signaling pathway [33], could significantly promote the proliferation in GC cells with restored CDO1, suggesting that NF-κB signaling as an antioxidant defense is probably activated in response to CDO1 re-expression. The potential relation of CDO1 to NF-κB signaling warrants further study.

To further delineate the mechanism by which CDO1-induced redox imbalance results in cytostasis, we turned our attention to ISR, since this signaling pathway can be activated by oxidative stress to block overall mRNA translation but increase the production of specific proteins, such as ATF4, in support of cellular viability [25]. A severe and prolonged stress, such as hyperosmotic status, induced stalling of mRNA translation and reduction in ATP production, as well as ISR to re-start cell cycle progression upon removal of pressure [34]. Similarly, as shown in our results, stable CDO1 expression also induced ISR via HRI, while Engeletin could alleviate oxidative stress and ISR in the same in vitro model. Moreover, ISR activation markedly inhibited the proliferation and, more importantly, barely triggered the death in GC cells. Notably, it has been unraveled recently that deregulated ISR in transformed cells or cancer-associated fibroblasts facilitated tumorigenesis and progression [35–37], thereby prompting development of the therapeutic strategy targeting ISR [38]. Interestingly, ISR could be activated through NF-κB signaling to promote the growth of the breast cancer cells that were refractory to endocrine therapy [39]. NF-κB signaling is a hub regulator of inflammation and gastric neoplasia [40, 41], thus whether NF-κB signaling is implicated in CDO1-induced ISR needs to be clarified.

There are some limitations in this study. First, an arising concern is that CDO1 probably plays distinct roles under specific conditions of GC, considering that it has been reported that activated ISR conferred resistance to cisplatin in GC cells [42] and our preliminary results have shown that patients with higher CDO1 expression had stronger resistance to the neoadjuvant chemotherapy SOX regimen (Data not shown). Present study just shows the inhibitory effect of CDO1 on the proliferation of the established GC cell lines, thereby the exact functions of CDO1 in GC initiation or therapy of patients with GC require further investigations. Second, how CDO1 remarkably enhances intracellular oxidative stress remains largely unknown, although SO3^2-^ formed Cys-SO_3_^2-^ to disturb GSH production. However, NAC failed to reduce oxidative stress in GC cells with re-expressed CDO1, implying that other mechanisms might be involved in this process. Thirdly, it is noteworthy that the survival result from Kaplan-Meier plotter (www.kmplot.com) was inconsistent with our findings (Fig. 1E). This contradiction was probably because we directly examined the potential correlation between patients’ prognosis and the protein product of CDO1 gene, which, in our opinion, could reveal more exact prognostic significance of CDO1. Additionally, one previous report validated that higher CDO1 gene methylation independently predicted worse prognosis in patients with gastric cancer [7]. However, further studies are needed to explain the discrepancy.

In conclusion, our data shows that CDO1 relies on its enzymatic activity to cause cytostasis in GC cells in vitro and in vivo, and the molecular mechanisms behind this phenotype is that CDO1 induces oxidative stress and subsequently ISR. Our results describe the anti-proliferation function of CDO1 in GC cells, and shed light on the possible intricate roles of CDO1 in GC pathology and therapy, which are needed to be fully evaluated.

## Materials and methods

### Clinical specimens

Human GC and matched non-tumor tissue samples were obtained from 130 patients receiving curative gastrectomy at Tianjin Medical University Cancer Hospital (Tianjin, China) from January 2004 to September 2007. These specimens were used in immunohistochemistry analysis of CDO1. Thirty pairs of GC and normal tissues were also collected from patients at Tianjin Medical University Cancer Hospital (Tianjin, China) in 2021, which were used in qPCR test of *CDO1* mRNA expression. All patients did not undergo neoadjuvant therapy.

### Cell culture

Human GC cell line NCI-N87 were purchased from the American Type Culture Collection (ATCC, USA). Human GC cell line MKN45 was a gift from Prof. Hui Li from Department of Gastrointestinal Cancer Biology at Tianjin Medical University Cancer Institute and Hospital, Tianjin, China. HEK-293T cells were generously provided by Prof. Zhihua Liu from the National Cancer Center/Cancer Hospital, Beijing, China. NCI-N87 and MKN45 cells were cultured in RPMI1640 supplemented with 10% fetal bovine serum (FBS). HEK-293T cells were cultured as recommended by ATCC. All cells with no >20 continuous passages were used in this study. All cell lines were verified as *Mycoplasma* negative.

### Antibodies and reagents

Primary antibodies against CDO1 (12589-1-AP), HRI (20582-1-AP), GADD34 (10449-1-AP), and Lamin B1 (12987-1-AP) were purchased from ProteinTech (Chicago, IL, USA). Antibodies against p-eIF2α (Ser51) (D9G8), eIF2α (D7D3), and Vinculin (E1E9V) were purchased from Cell Signaling Technology (Danvers, MA, USA). Anti-ATF4 antibody (ab184909) was purchased from Abcam (Cambridge, England).

Puromycin dihydrochloride (HY-B1743A), JC-1 (HY-15534), antioxidant compound library (HY-L037), Engeletin (HY-N0436), ISRIB (HY-12495), and Cell Counting Kit-8 (CCK-8, HY-K0301) were purchased from MedChemExpress (Shanghai, NJ, USA). Engeletin (100 mM) and ISRIB (10 mM) were solved using DMSO respectively according to the instruction of the manufacturer. The final concentration of Engeletin (5 μM) and ISRIB (10 μM) were used in the experiments.

### Plasmids, lentivirus production, and generation of stable cell lines

The full-length cDNA of human CDO1 (CDO1^WT^) was cloned into the pLVX-IRES-puro vector. To abrogate the enzymatic activity of CDO1, we replaced the tyrosine residue with phenylalanine at CDO1 (CDO1^Y157F^) [19]. Human eIF2α cDNA (eIF2α^WT^) was engineered into pLVX-IRES-neo vector, and the serine residue was mutated to alanine (eIF2α^S51A^) to eliminate the activation of ISR. Additionally, human GADD34 cDNA was cloned into pLVX-IRES-neo vector. Empty pLVX-IRES-puro or pLVX-IRES-neo vector was used as the negative control (N.C.). Lentivirus was produced by simultaneously introducing pLVX-IRES-puro/neo, pMD2.G (Plasmid #12259, Addgene), and psPAX2 (Plasmid #12260, Addgene) into HEK-293T cells. The detailed procedures of lentivirus package and collection were described as previously [43]. NCI-N87 and MKN45 cells were infected with the indicated lentiviruses, and stable cell populations were established using puromycin or/and G418. The gene expression efficacy was measured via immunoblots.

### siRNA and transfection

siRNAs were synthesized by RiboBio (Guangzhou, China), and the sequences are listed in Supplementary Table 1. A pool of the three separate siRNAs, which were designed to target a single gene, were delivered into NCI-N87 or MKN45 cells using lipofectamine 2000, and the final concentration of siRNAs was 100 nM. A scramble siRNA (100 nM) was used as the negative control (si-N.C.).

### Cellular proliferation assay in vitro

CCK-8 assays were performed to examine cell proliferation in vitro. Briefly, a total of 1000 MKN45 cells or 3000 NCI-N87 cells were seeded into 96-well plates in sextuple. The absorbance at 450 nm was measured by a microplate reader (BioTek) at the indicated time points. For EdU incorporation assays, replicating MKN45 or NCI-N87 cells were evaluated using the Click-iT™ EdU Alexa Fluor™ 488 Flow Cytometry Assay Kit (Thermo Fisher Scientific). For colony formation assay, NCI-N87 cells (5 × 10^3^ cells/well) from the control and the CDO1-restored group were seeded in 6-well plates respectively and cultured for around 10 days. The colonies were fixed with methanol for 15 min, stained with 0.5% crystal violet, and counted under microscope.

### ROS, ATP, and GSH/GSSG measurement

Intracellular ROS levels were measured using ROS-Glo™ H_2_O_2_ Assay Kit (Promega, Madison, WI, USA). Intracellular ATP levels were detected using CellTiter-Glo Luminescent Cell Viability Assay Kit (Promega). The alterations in intracellular GSH/GSSG levels were examined using GSH/GSSG-Glo™ Assay Kit (Promega). All experiments were performed according to the manufacturer’s instruction. The luminescent intensity was measured via a microplate reader (BioTek) at the indicated time points.

### Measurement of glucose uptake and lactate secretion in vitro

The level of glucose intake from culture medium and of lactic acid production in N.C. and CDO1-overexpressing MKN45 or NCI-N87 cells were detected using Glucose Uptake-Glo™ kit (Promega) and Lactate-Glo™ kit (Promega) respectively, according to the instructions of the manufacturer.

### RNA extraction and RT-qPCR

Total RNA was extracted from cultured cells or GC/normal tissue samples using RNAiso plus (Takara Bio, Shiga, Japan). The cDNAs were generated using GoScript™ Reverse Transcription Kit (Promega). The mRNA levels of CDO1 were tested using TB Green Premix Ex TaqTM II (Takara Bio) on the QuantStudio 5 real-time PCR system (Applied Biosystems, Foster City, CA, USA). GAPDH was used for data normalization. The − ΔCt method was used to determine CDO1 expression in tissues, and the 2^−ΔΔCt^ method was used to evaluate the expression of the indicated genes in GC cell lines. The qPCR primer sequences in this study were listed in Supplementary Table 2.

### Immunoblots

The total proteins from the indicated cells were extracted using lysis buffer (10 mM Tris-HCl, 150 mM NaCl, 5 mM EDTA, 1% Triton X-100, 0.25% sodium deoxycholate, pH = 7.4) supplemented with protease and phosphatase inhibitors (Roche). The protein concentrations were determined using Pierce^TM^ BCA protein assay kit (Thermo Fisher Scientific). Immunoblotting assays were performed as described previously [43]. The images were acquired using Amersham Imager 600 System (GE). Original western blots of the representative results were presented in Supplementary File 1.

### Immunofluorescence

JC-1 was dissolved in DMSO as a stocking solution (200 μM). For immunofluorescence assay, MKN45 and NCI-N87 cells were seeded in 96-well black/clear bottom plates (Corning, NY, USA). Add JC-1 (200 μM) to each well to make the final concentration at 2 μM. The cells were incubated with JC-1 for 15 min and washed twice with PBS. Then the cells were observed and images were acquired using Opera Phenix High-Content Screening System (PerkinElmer, Waltham, MA, USA). The ratio of red/green fluorescence was analyzed via Harmony^®^ high-content analysis software (PerkinElmer).

### Immunohistochemistry (IHC)

The human GC/normal tissue samples were stained with anti-CDO1 antibody (1:400) according to the procedures described before [43]. Positive cells and staining intensity were scored separately. Then, the IHC staining score was calculated. The cytoplasmic expression of CDO1 was assessed by assigning scores to the average intensity of positive tumor cells.

### Targeted metabolomics study

The control or CDO1-restored MKN45 cells were seeded in 10-cm plates and collected using cell scrapers 24 h later. The collected cells were immediately frozen using liquid nitrogen and stored in a − 80 °C freezer. The cell samples were then subjected to liquid chromatography–mass spectrometry (LC–MS) analysis of 32 critical metabolites implicated in energy metabolism by Applied Protein Technology (Shanghai, China) (Supplementary Table 3). Briefly, the cell samples were vortexed in 1 ml methanol:acetonitrile:H_2_O (2:2:1, v/v), sonicated for 4 min, and incubated at −20 °C for 1 h to remove proteins. Agilent 1260 instrument was employed for high performance liquid chromatography, as the mobile Phase A was H_2_O plus 25 mM ammonium acetate and 25 mM ammonia (pH = 9.75) and the mobile Phase B was acetonitrile. Agilent 6460 QqQ mass spectrometer was used for detection. The operating parameters were set as follows: sheath gas temperature, 350 °C; dry gas temperature, 350 °C; sheath gas flow, 11 l/min; dry gas flow, 10 l/min; capillary voltage, 4,000 V or −3,500 V in positive or negative modes, respectively; nozzle voltage, 500 V; and nebulizer pressure, 30 psi. The dwell time for each MRM transition was 3 msec, and the total cycle time was 1.263 sec. The data was analyzed using the MRMAnalyzer.

### High-throughput compound screening

The antioxidant compound library containing 778 chemicals was used for high-throughput screen (HTS) of specific antioxidants to alleviate the oxidative stress caused by CDO1 expression. MKN45 cells with restored CDO1 were seeded in 96-well white plates (Corning). After 24 h incubation, the cells attained around 50% confluence. The drugs were then added into culture medium using Explorer G3 Integrated Workstation (PerkinElmer) and incubated for 24 h in a humidified CO_2_ (5%) chamber at 37 °C. The final concentration of each drug was 5 μM. MKN45 cells treated with DMSO acted as the negative control. Intracellular ATP level, as the surrogate of cellular viability, was examined using CellTiter-Glo luminescent reagent (Promega) on EnVision Microplate Reader equipped with HTS mode (PerkinElmer). The luminescent intensity of the cells treated with each drug was compared to that of the control cells, and the fold changes were displayed as heatmaps. The fold changes were measured as: Fold change = (Reads _(antioxidant)_-Reads _(Vehicle)_) / Reads _(Vehicle)_.

### Tumor xenografts in nude mice

Five-week-old female Balb/c nude mice were purchased from Vital River Laboratories (Beijing, China) and housed under specific-pathogen-free (SPF) conditions. To test the in vivo growth of GC cells, 5.0 × 10^5^ MKN45 cells or 1.5 × 10^6^ NCI-N87 cells from the indicated groups were inoculated subcutaneously into the Balb/c nude mice. The tumors were measured every 3 days, and the tumor volume was calculated as: *V* = length × width^2^ × 0.5. Mice were sacrificed at the 20th day after the inoculation and the harvested tumors were weighed.

### Statistical analysis

Except animal, IHC and HTS assays, all experiments were performed at least twice independently, and all values are expressed as mean ± standard deviation. GraphPad Prism version 8 (San Diego, CA, USA) was used, and tests were performed using Student’s *t*-test or *χ*^2-^test unless otherwise specified. *p* < 0.05 was considered statistically significant. Significance levels *p < 0.05; **p < 0.01; ***p < 0.001.

## Supplementary information

Supplementary Figures

Supplementary table 1

Supplementary table 2

Supplementary table 3

Supplementary file 1

TR test of MKN45

STR test of NCI-N87

## Data Availability

The GC datasets (GSE29272) from the GEO repository database (https://www.ncbi.nlm.nih.gov/gds) was used in this study. The data that support the findings of this study are available from the corresponding author upon reasonable request.
