# Supplementary material for: Cysteine dioxygenase 1 attenuates the proliferation via inducing oxidative stress and integrated stress response in gastric cancer cells
Source: Cell Death Discov. 2022 Dec 16;8:493. doi: 10.1038/s41420-022-01277-x (PMC9758200; doi:10.1038/s41420-022-01277-x)
Supplement: Supplementary file 1 — Supplementary Figures [file 41420_2022_1277_MOESM1_ESM.docx]

**Supplementary Figures**

**Cysteine dioxygenase 1 attenuates the proliferation via inducing oxidative stress and integrated stress response in gastric cancer cells**

Gang Ma^1,^ *, Zhenzhen Zhao^1,^ *, Yang Qu^2^, Fenglin Cai^1^, Siya Liu^1^, Han Liang^1^, Rupeng Zhang^1^, Jingyu Deng^1^

1. Department of Gastric Surgery, Tianjin Medical University Cancer Institute and Hospital, National Clinical Research Center for Cancer; Key Laboratory of Cancer Prevention and Therapy, Tianjin; Tianjin's Clinical Research Center for Cancer, Tianjin, 300060, P. R. China

2. Department of Gastrointestinal Cancer Biology, Tianjin Medical University Cancer Institute and Hospital, National Clinical Research Center for Cancer; Key Laboratory of Cancer Prevention and Therapy, Tianjin; Tianjin's Clinical Research Center for Cancer, Tianjin, 300060, P. R. China

* These authors contributed equally to this work.

Corresponding author: Pro. Jingyu Deng, dengery@126.com.

**Supplementary Figures and Figure Legends:**


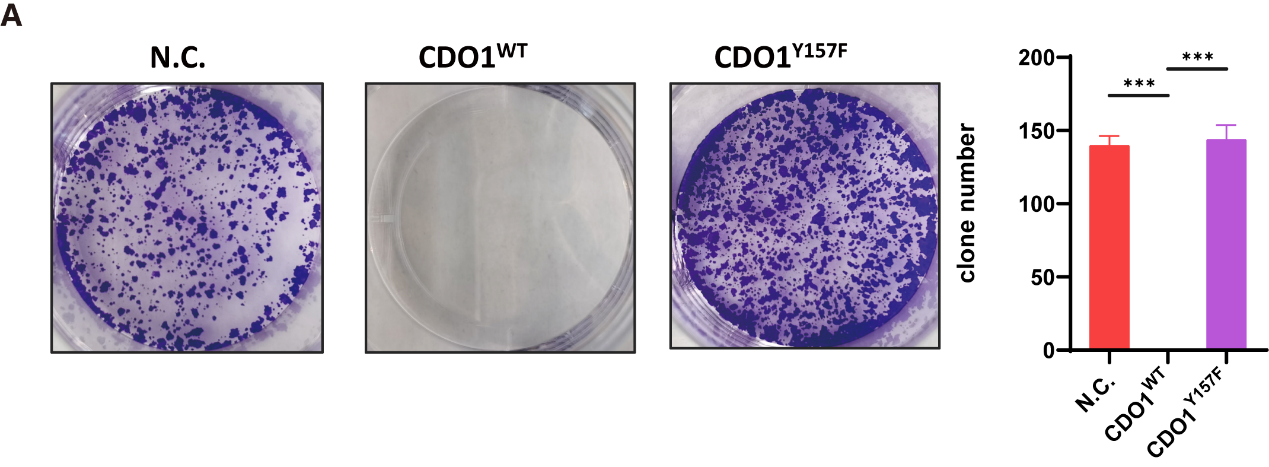


**Supplementary Fig. 1** Colony formation assay was conducted to test the proliferation of NCI-N87 cells from the indicated groups, showing that the proliferative potential of NCI-N87 cells with CDO1^Y157F^ was reminiscent of that in control NCI-N87 cells. The experiments were performed twice independently, and one representative result is presented here. ***p < 0.001.


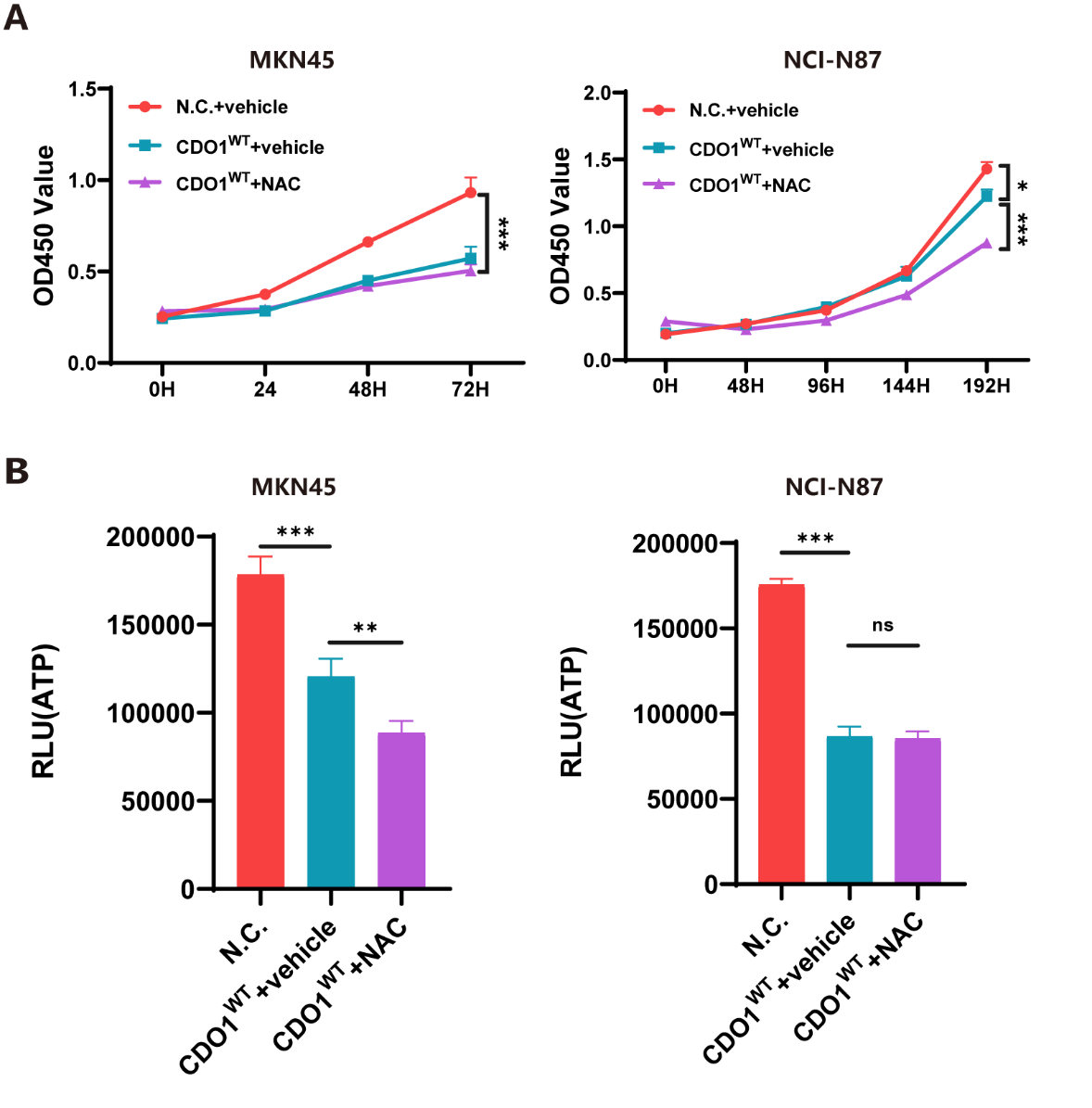


**Supplementary Fig. 2** NAC treatment does not significantly relieve the inhibitory effect of CDO1 on the proliferation in GC cells. **(A)** CCK-8 assays showed that NAC-treated MKN45 (left panel) and NCI-N87 (right panel) cells did not grew faster than vehicle-exposed ones at the indicated time points (N = 6, mean ± SD), when CDO1^WT^ was overexpressed in both cell populations. One representative result is presented here. **(B)** ATP production in NAC-treated MKN45 (left panel) and NCI-N87 (right panel) cells with restored CDO1^WT^ was not more than that in vehicle-treated cells. One representative result is presented here. n.s. means no significance, *p < 0.05, **p < 0.01, ***p < 0.001.


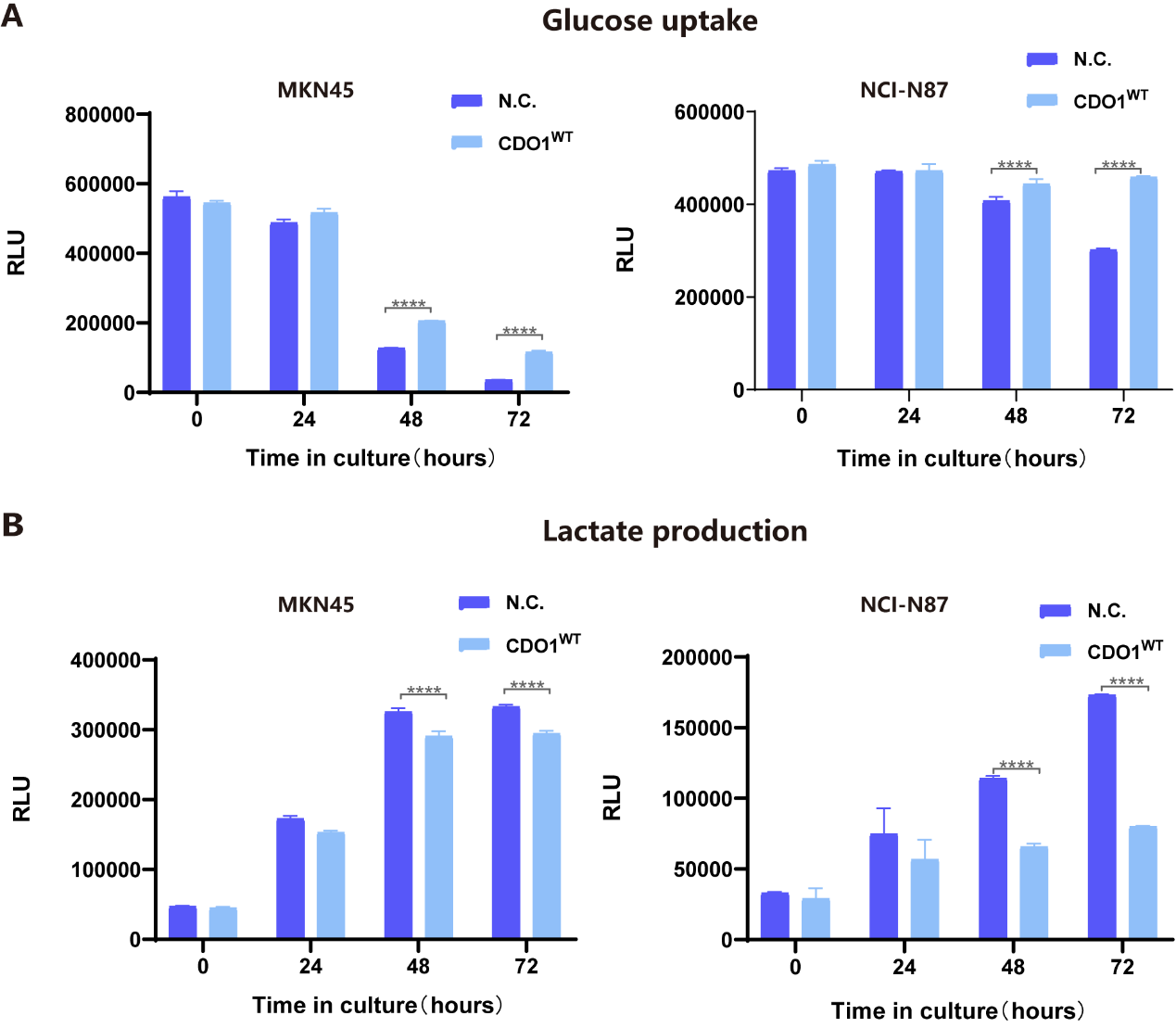


**Supplementary Fig. 3 (A)** CDO1^WT^ decreased the glucose uptake in MKN45 (left panel) and NCI-N87 (right panel) cells, as compared with those control counterparts. **(B)** CDO1^WT^ reduced the production of lactate in MKN45 (left panel) and NCI-N87 (right panel) cells. ****p < 0.0001.


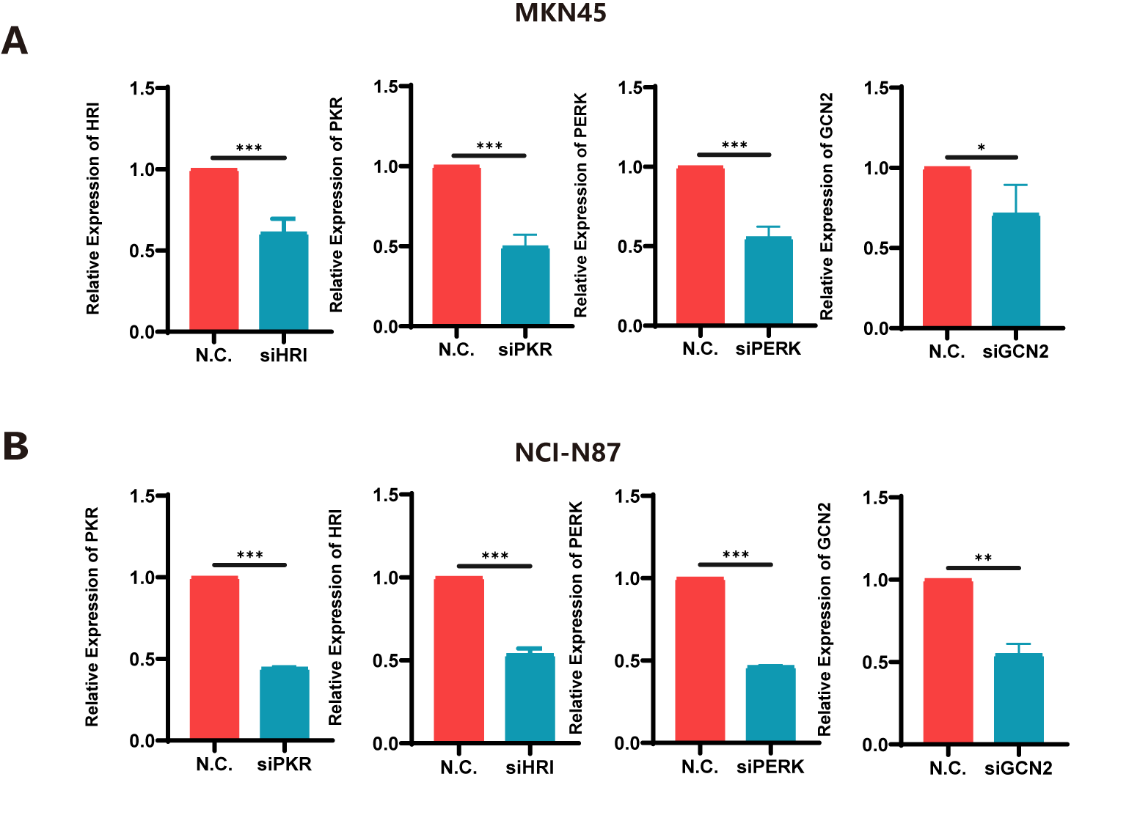


**Supplementary Fig. 4** qPCR test was used to determine the knockdown efficiency of siRNAs against HRI, PKR, PERK, and GCN2 in MKN45 (upper panel) (A) and NCI-N87 (bottom panel) (B) cells. *p < 0.05, **p < 0.01, ***p < 0.001.


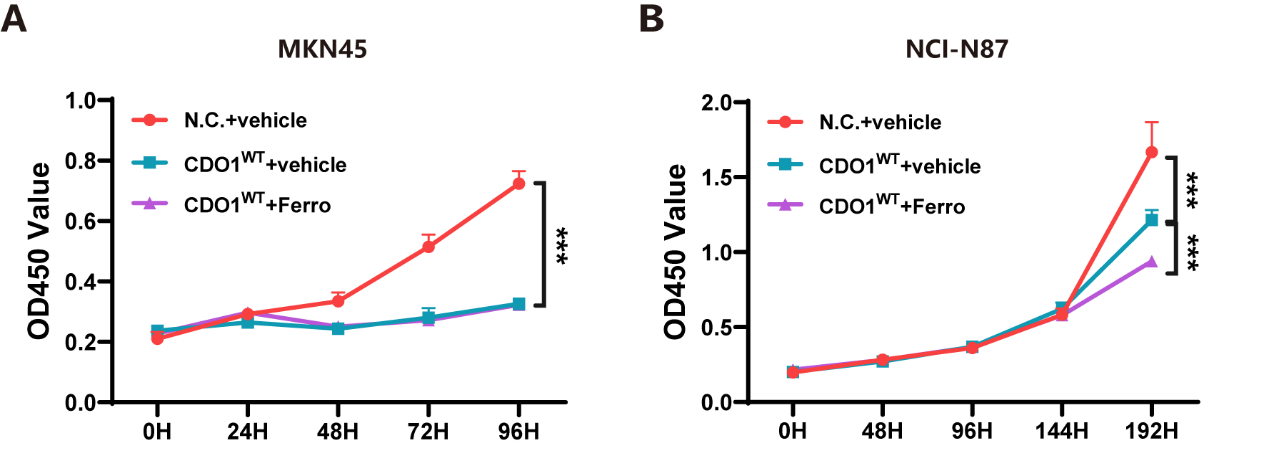


**Supplementary Fig. 5** CCK-8 assays showed that CDO1^WT^ did not induce ferroptosis in GC cells, as indicated by CCK-8 assays that Ferrostatin-1, one commonly used inhibitor of ferroptosis, failed to restore the proliferation in MKN45 (left panel) and NCI-N87 (right panel) cells.
