# Supplementary material for: Cysteine dioxygenase 1 attenuates the proliferation via inducing oxidative stress and integrated stress response in gastric cancer cells
Source: Cell Death Discov. 2022 Dec 16;8:493. doi: 10.1038/s41420-022-01277-x (PMC9758200; doi:10.1038/s41420-022-01277-x)
Supplement: Supplementary file 2 — Supplementary table 1 [file 41420_2022_1277_MOESM2_ESM.docx]

Supplementary Table 1 siRNAs against HRI, PKR, PERK and GCN2

| **Target Gene** | **siRNA1** | **siRNA2** | **siRNA3** |
| --- | --- | --- | --- |
| **HRI** | GCACAAACTTCACGTTACT | AGAGCAATGTGGTGTTAAA | CCAGTTCAATTGTGGAACA |
| **PKR** | GGGATGGCATCATCTCAGA | GCGAGAAACTAGACAAAGT | GGGATGGATTTGATTATGA |
| **PERK** | GGAACGACCTGAAGCTATA | GGAAACAGCTATTCTCATA | GCATCTGCCTGGTTACTTA |
| **GCN2** | GGACTAAAGTCACTGATGA | CCATCAACCTAACCCAGAA | GCAATTCTGTGGTGCATAA |
