# Supplementary material for: Cysteine dioxygenase 1 attenuates the proliferation via inducing oxidative stress and integrated stress response in gastric cancer cells
Source: Cell Death Discov. 2022 Dec 16;8:493. doi: 10.1038/s41420-022-01277-x (PMC9758200; doi:10.1038/s41420-022-01277-x)
Supplement: Supplementary file 3 — Supplementary table 2 [file 41420_2022_1277_MOESM3_ESM.docx]

Supplementary Table 2 qPCR primers used in this study

| **Primer Name** | **Forward Primer Sequence (5' to 3')** | **Reverse Primer Sequence (5' to 3')** |
| --- | --- | --- |
| **CDO1** | TCCATTGGCTTACATCGAGTAGA | CCCGAAGTTGCATTTGGAGT |
| **ATF3** | CGCTGGAATCAGTCACTGTCAG | CTTGTTTCGGCACTTTGCAGCTG |
| **ATF4** | TTCTCCAGCGACAAGGCTAAGG | CTCCAACATCCAATCTGTCCCG |
| **TRIB3** | GCTTTGTCTTCGCTGACCGTGA | CTGAGTATCTCAGGTCCCACGT |
| **GADD34** | TCCGACTGCAAAGGCGGCTCA | CAGCCAGGAAATGGACAGTGAC |
| **GAPDH** | TGCACCACCAACTGCTTAGC | GGCATGGACTGTGGTCATGAG |
