# Supplementary material for: Cysteine dioxygenase 1 attenuates the proliferation via inducing oxidative stress and integrated stress response in gastric cancer cells
Source: Cell Death Discov. 2022 Dec 16;8:493. doi: 10.1038/s41420-022-01277-x (PMC9758200; doi:10.1038/s41420-022-01277-x)
Supplement: Supplementary file 4 — Supplementary table 3 [file 41420_2022_1277_MOESM4_ESM.docx]

Supplementary Table 3 Metabolites detected in the targeted metabolic profile

| **Metabolites** | **Energy pathway** |
| --- | --- |
| 3-Phospho-D-glycerate | glycolysis |
| Acetyl coenzyme A (Acetyl-CoA) | TCA |
| Adenosine 5'-triphosphate (ATP) | TCA/glycolysis/oxidative phosphorylation |
| ADP | TCA/glycolysis/oxidative phosphorylation |
| Alpha-Ketoglutarate | TCA |
| AMP | TCA/glycolysis |
| Beta-D-Fructose 6 phosphate (β-D-F6P) | glycolysis |
| cis-Aconitate | TCA |
| cyclic-AMP | AMP derivative |
| D-Fructose 1,6-biphosphate | glycolysis |
| D-Glucose-6-phosphate (G6P) | glycolysis |
| Dihydroxyacetone phosphate (DHAP) | glycolysis |
| Flavin mononucleotide(FMN) | oxidative phosphorylation |
| Fumarate/Fumaric acid | TCA |
| GMP | pentose phosphate |
| Guanosine 5'-diphosphate (GDP) | TCA |
| Guanosine -5'-tridiphosphate (GTP) | TCA |
| Isocitrate | TCA |
| Lactate | glycolysis |
| L-Malate acid | TCA |
| NAD+ | oxidative phosphorylation |
| NADP+ | pentose phosphate |
| NADPH | pentose phosphate |
| Oxaloacetate | TCA |
| Phosphoenolpyruvate (PEP) | glycolysis |
| Pyruvate/Pyruvic acid | TCA |
| Reduced nicotinamide adenine dinucleotide (NADH) | oxidative phosphorylation |
| Succinate/Succinic acid | TCA |
| Succinyl-CoA | TCA |
| Thiamine pyrophosphate (TPP) | TCA |
| citrate | TCA |
| Glutaryl-CoA | Tryptophan metabolism |
