# Supplementary material for: Cysteine dioxygenase 1 attenuates the proliferation via inducing oxidative stress and integrated stress response in gastric cancer cells
Source: Cell Death Discov. 2022 Dec 16;8:493. doi: 10.1038/s41420-022-01277-x (PMC9758200; doi:10.1038/s41420-022-01277-x)
Supplement: Supplementary file 5 — Supplementary file 1 [file 41420_2022_1277_MOESM5_ESM.pptx]

## Slide 1
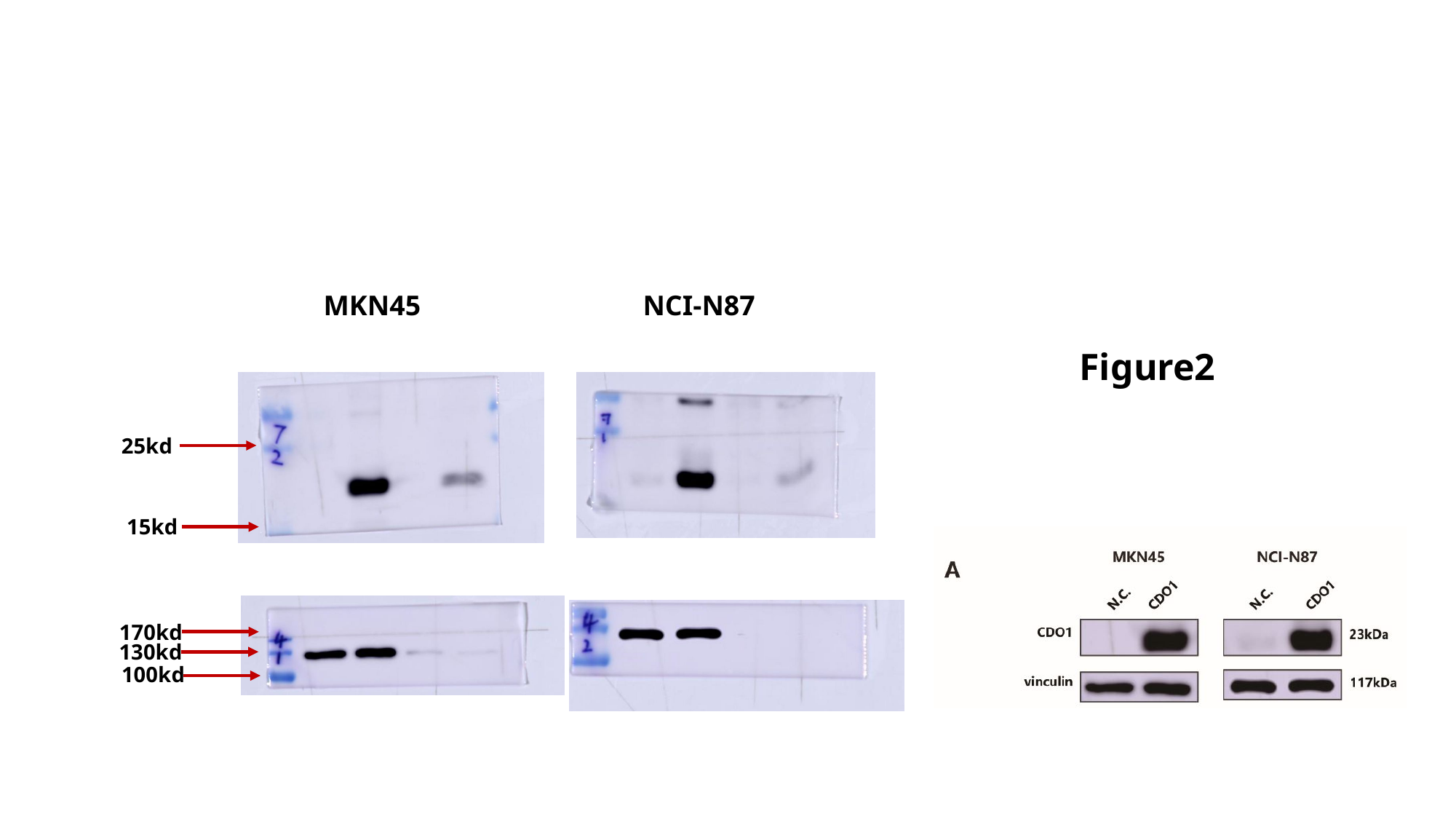

MKN45
NCI-N87
Figure2
25kd
15kd
170kd
130kd
100kd

## Slide 2
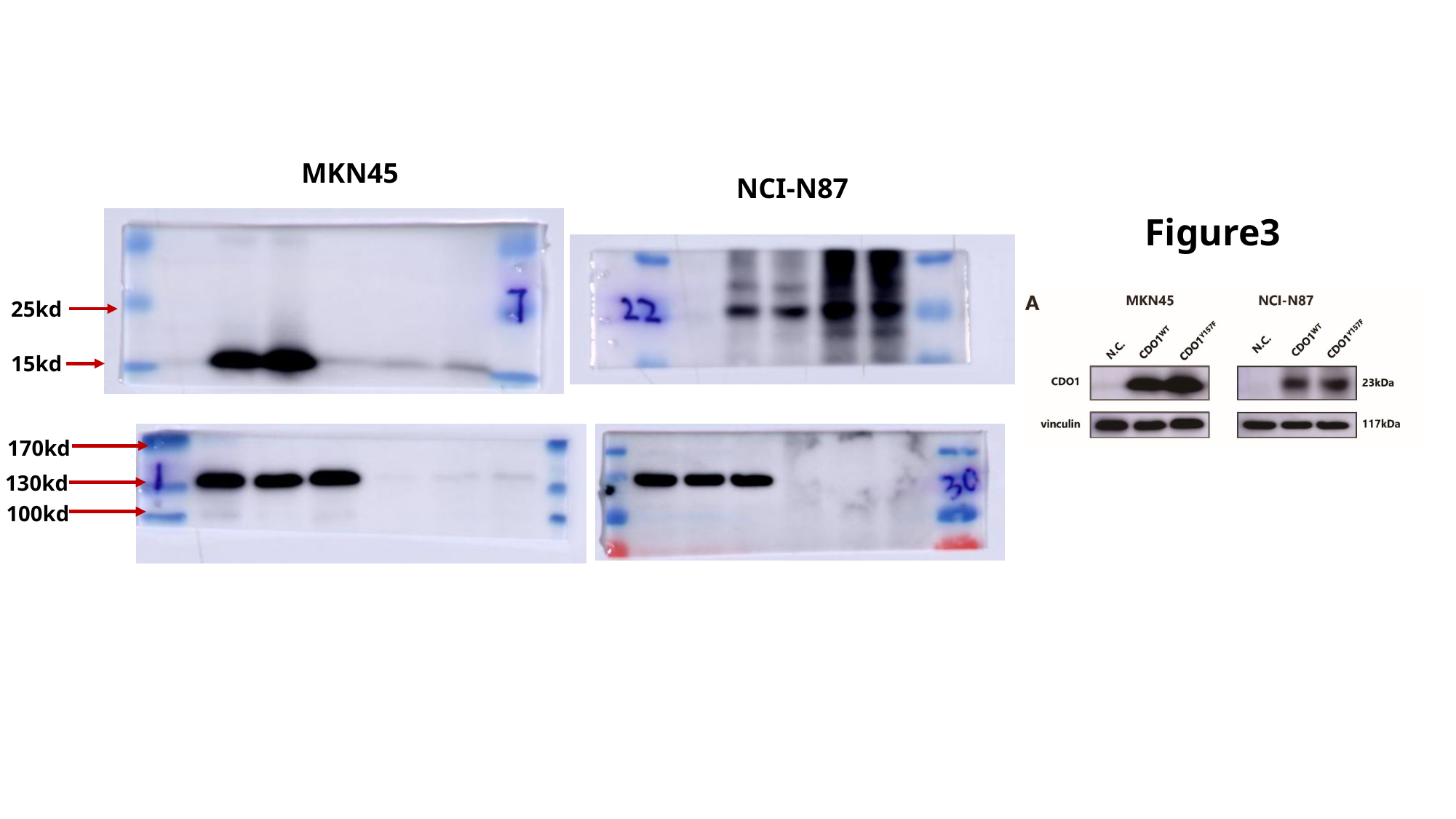

MKN45
NCI-N87
Figure3
25kd
15kd
170kd
130kd
100kd

## Slide 3
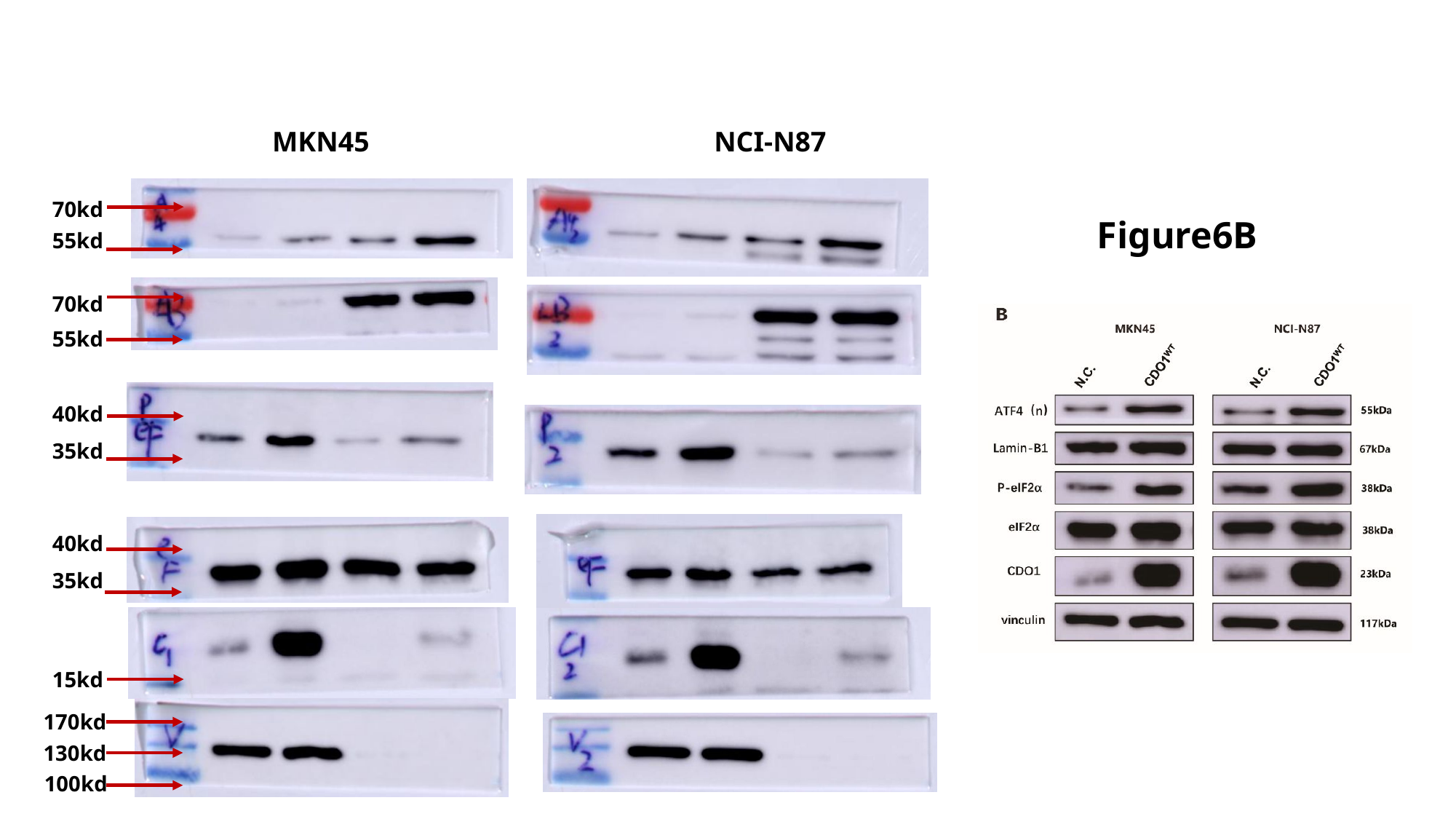

MKN45
NCI-N87
70kd
Figure6B
55kd
70kd
55kd
40kd
35kd
40kd
35kd
15kd
170kd
130kd
100kd

## Slide 4
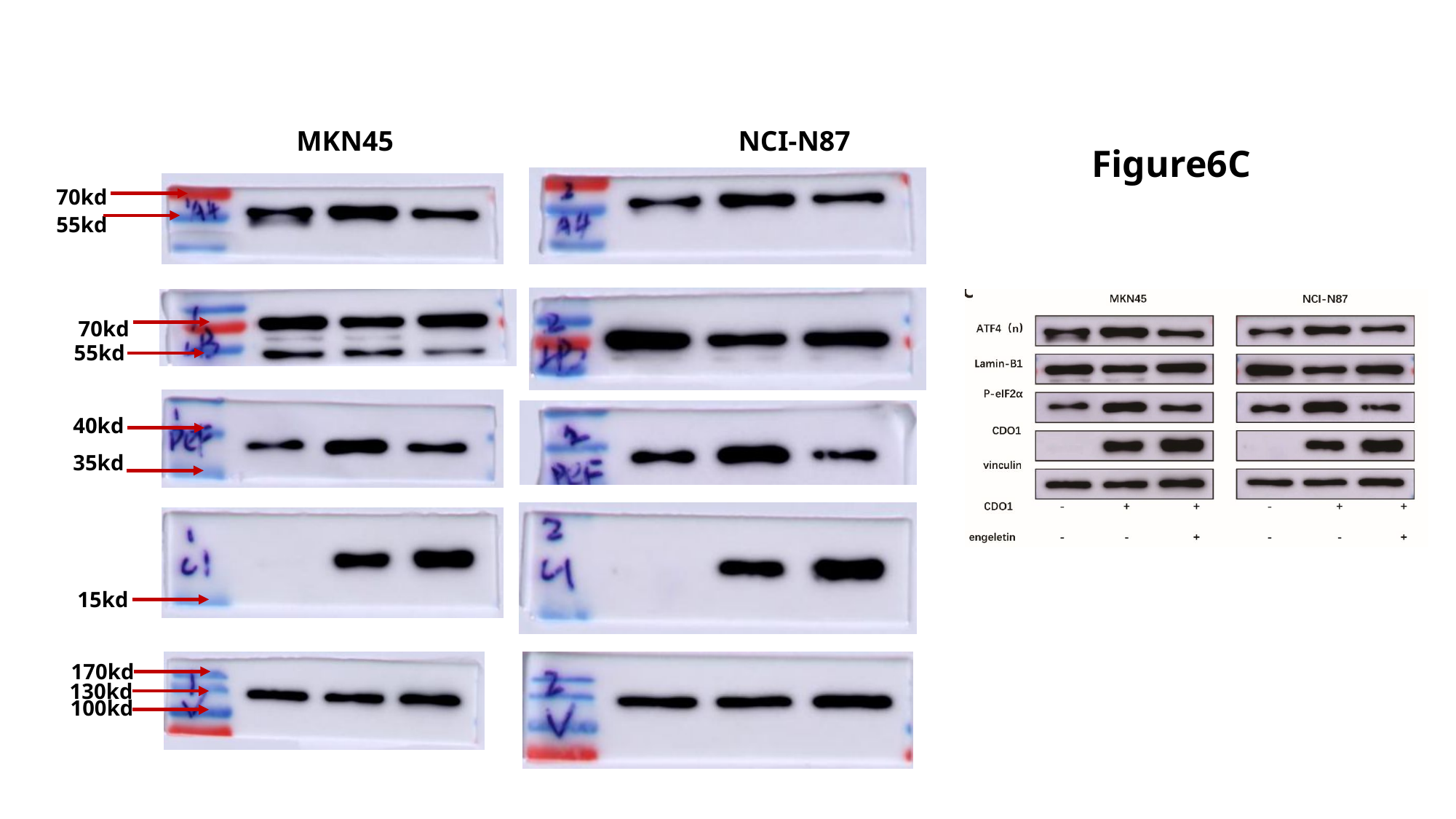

MKN45
NCI-N87
Figure6C
70kd
55kd
70kd
55kd
40kd
35kd
15kd
170kd
130kd
100kd

## Slide 5
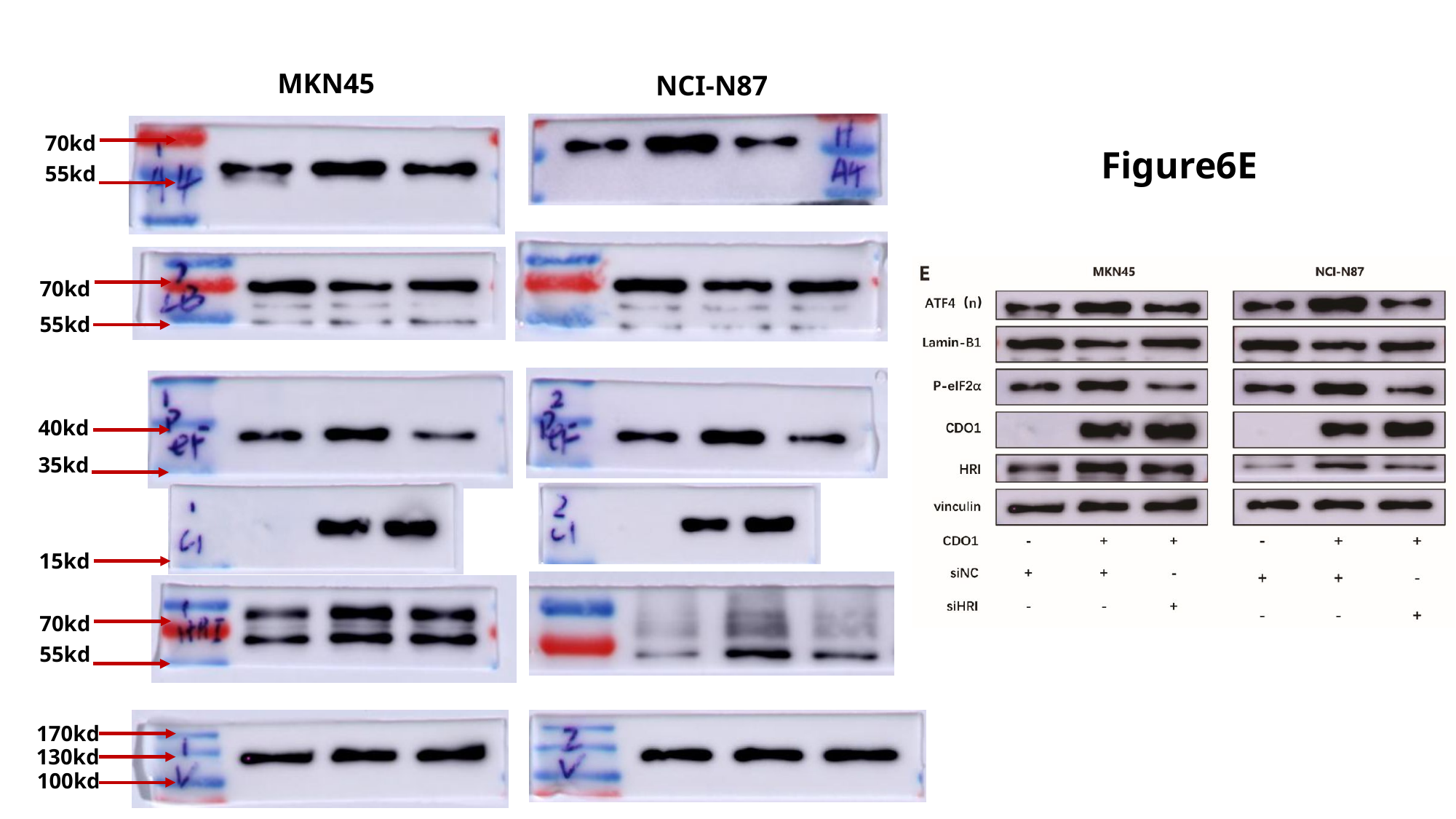

MKN45
NCI-N87
70kd
Figure6E
55kd
70kd
55kd
40kd
35kd
15kd
70kd
55kd
170kd
130kd
100kd

## Slide 6
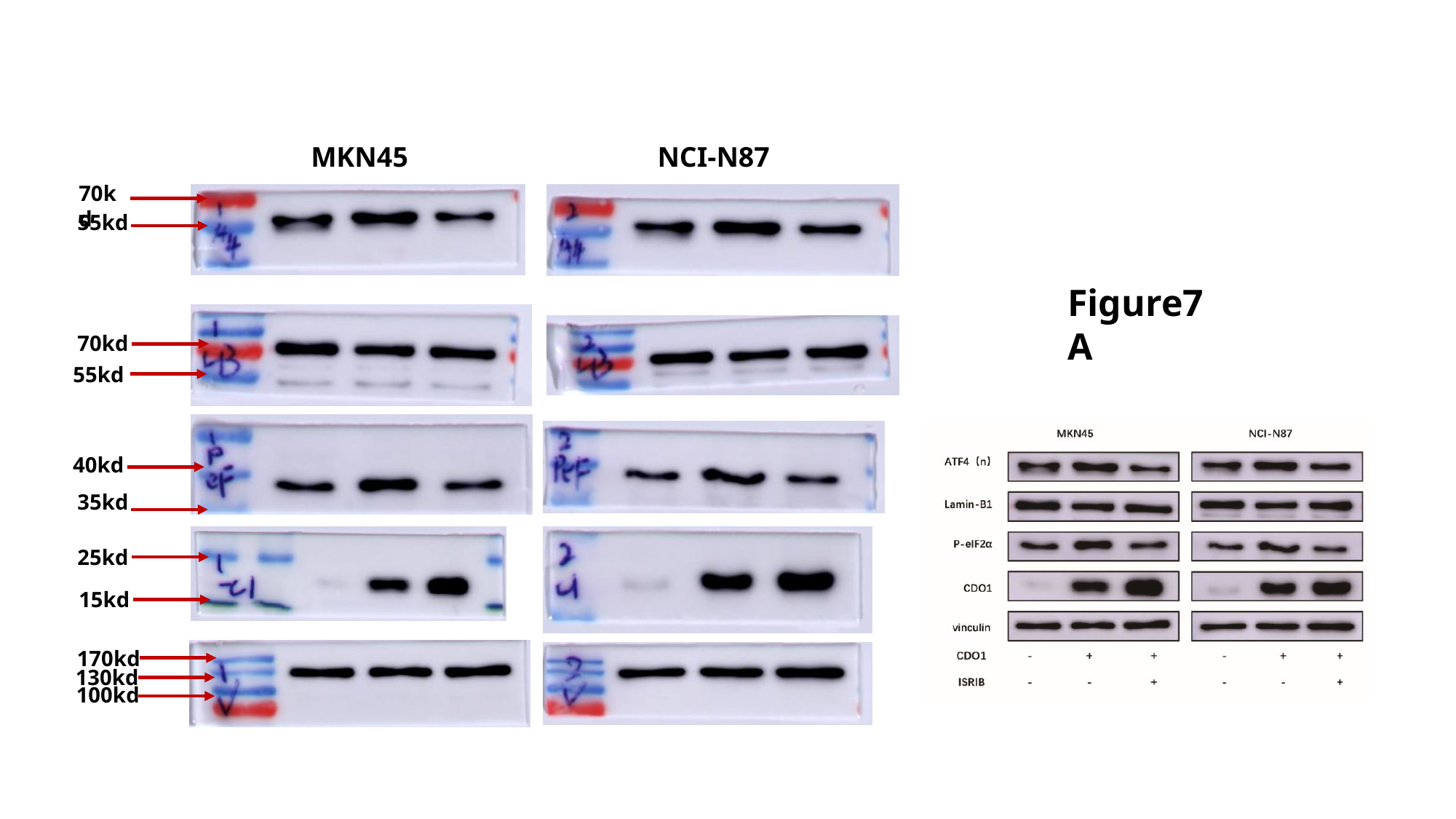

MKN45
NCI-N87
70kd
55kd
Figure7A
70kd
55kd
40kd
35kd
25kd
15kd
170kd
130kd
100kd

## Slide 7
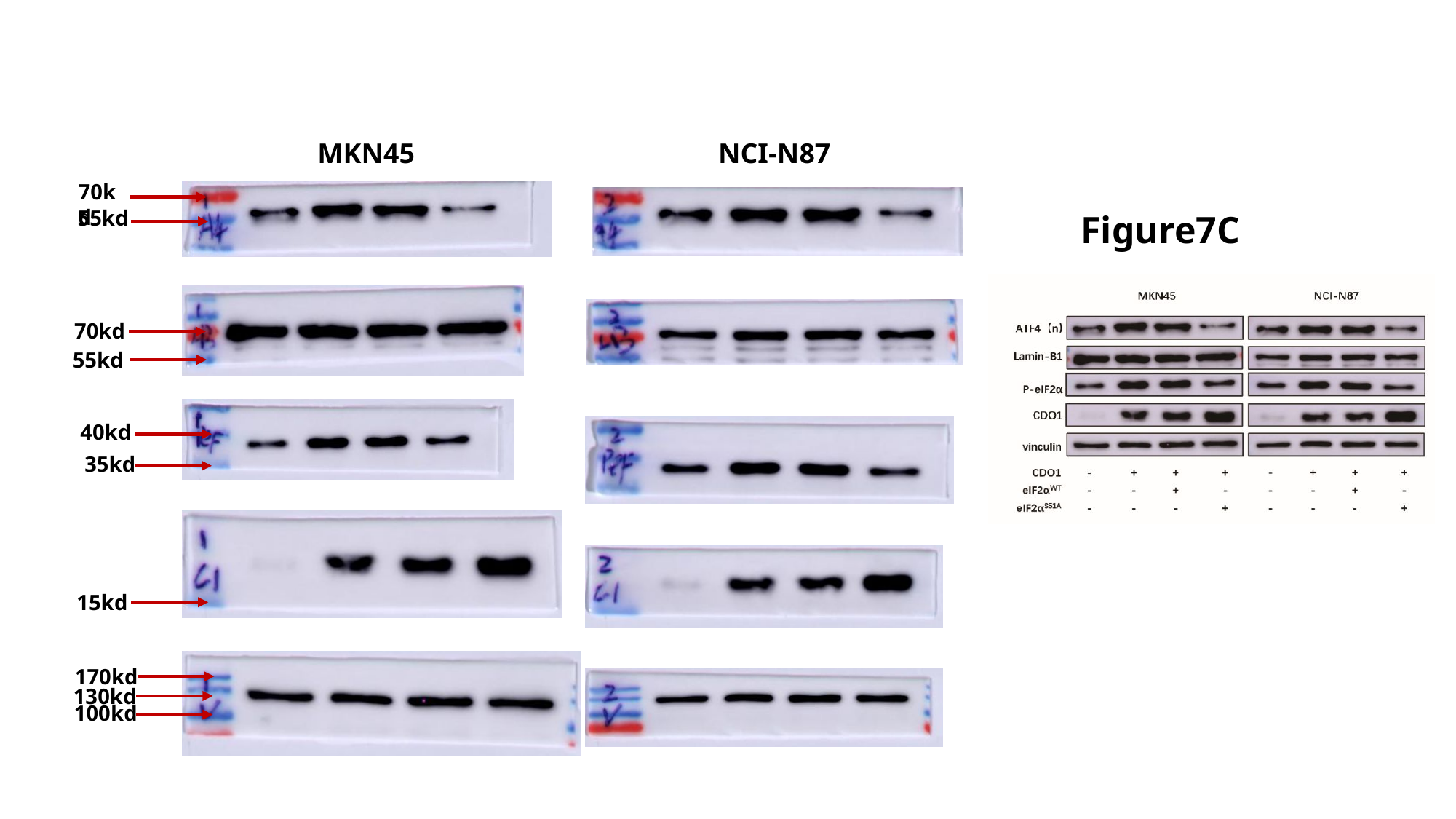

MKN45
NCI-N87
70kd
55kd
Figure7C
70kd
55kd
40kd
35kd
15kd
170kd
130kd
100kd

## Slide 8
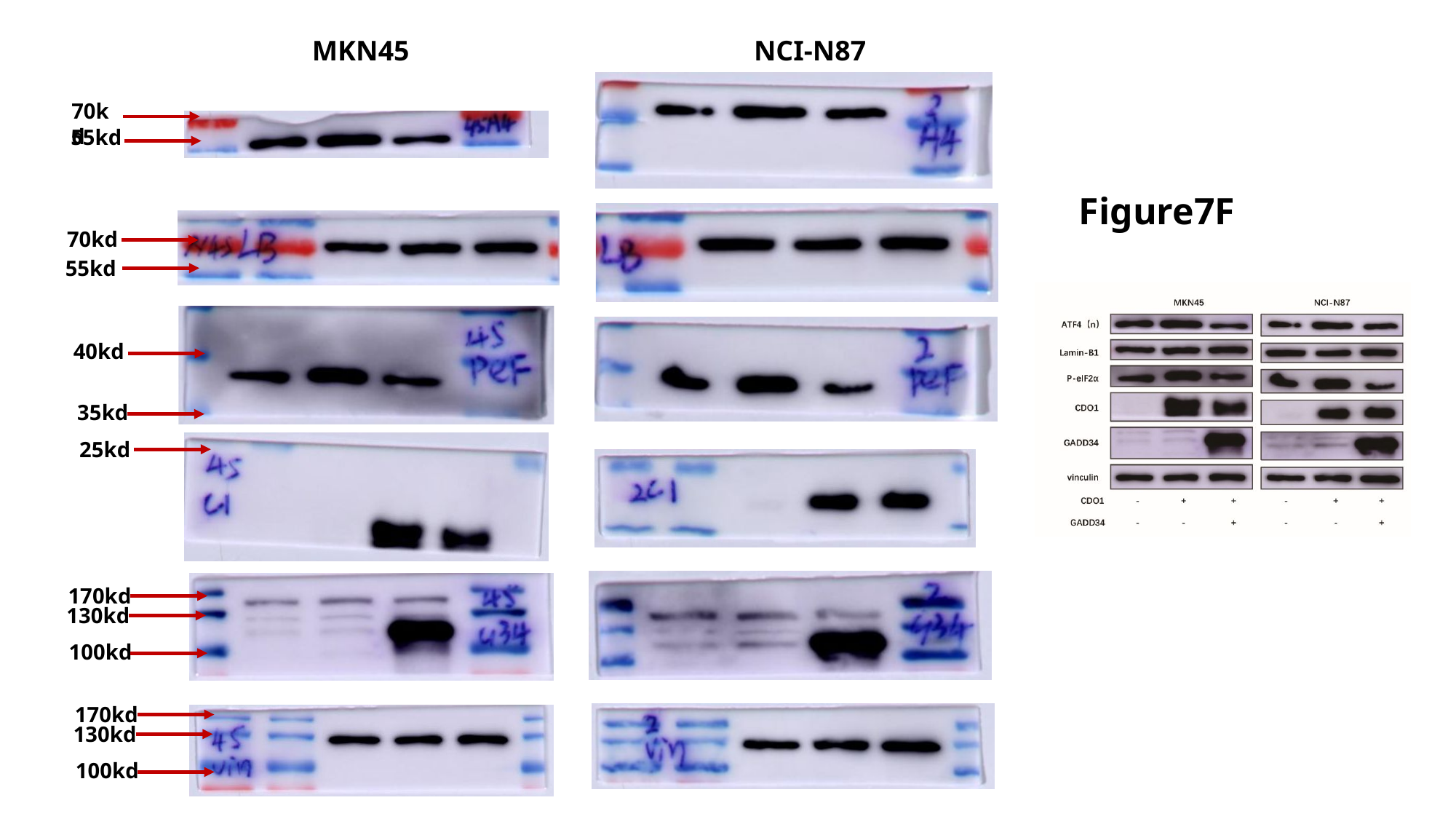

MKN45
NCI-N87
70kd
55kd
Figure7F
70kd
55kd
40kd
35kd
25kd
170kd
130kd
100kd
170kd
130kd
100kd
