# Supplementary figures and images for: Cysteine dioxygenase 1 attenuates the proliferation via inducing oxidative stress and integrated stress response in gastric cancer cells
Source: Cell Death Discov. 2022 Dec 16;8:493. doi: 10.1038/s41420-022-01277-x (PMC9758200; doi:10.1038/s41420-022-01277-x)

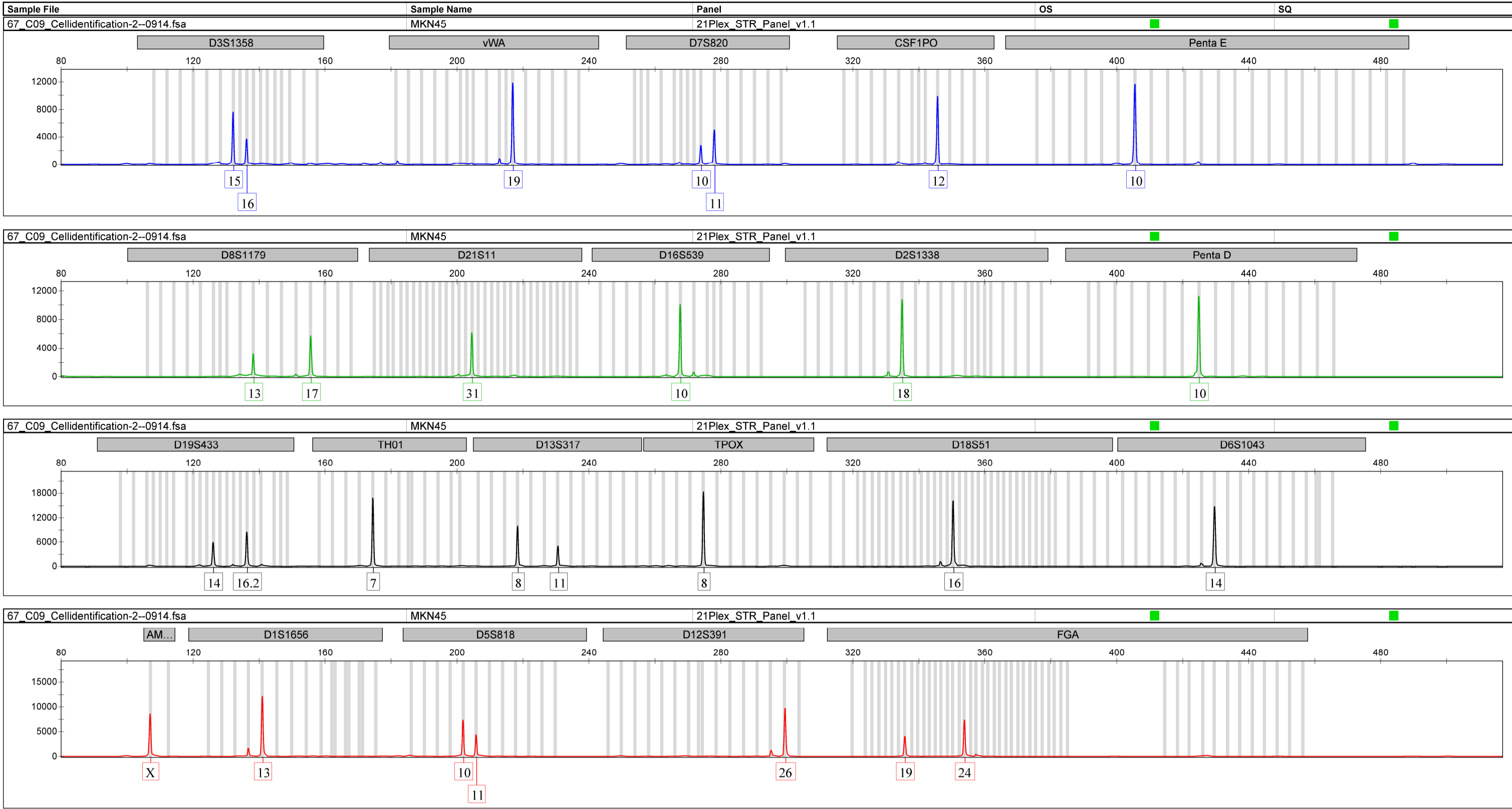

Supplement: Supplementary file 6 — TR test of MKN45 [file 41420_2022_1277_MOESM6_ESM.pdf]

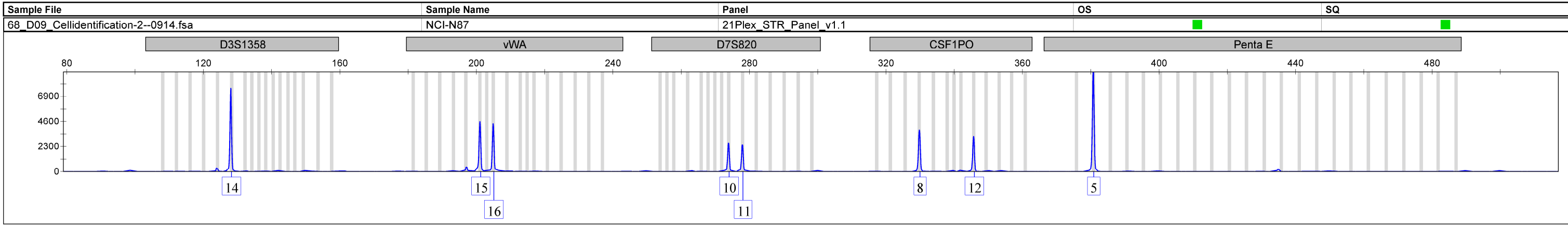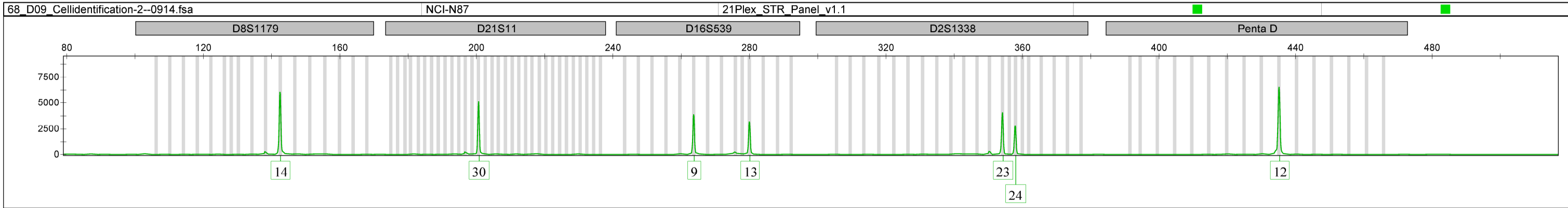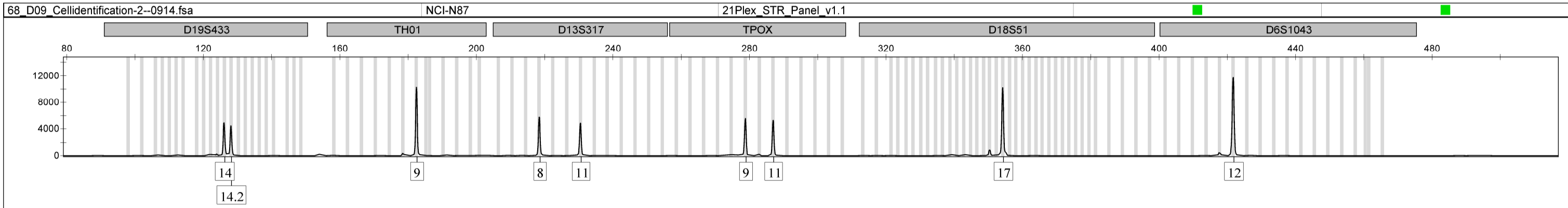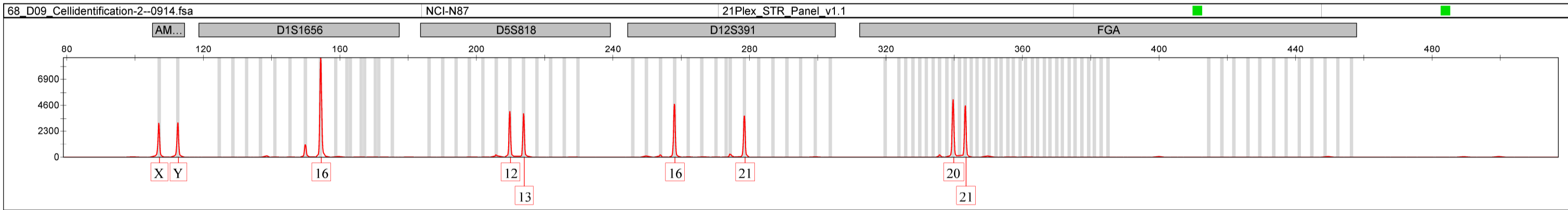

Supplement: Supplementary file 7 — STR test of NCI-N87 [file 41420_2022_1277_MOESM7_ESM.pdf]
